# Supplementary material for: Annular Tautomerism of 3(5)-Disubstituted-1H-pyrazoles with Ester and Amide Groups
Source: Molecules. 2019 Jul 19;24(14):2632. doi: 10.3390/molecules24142632 (PMC6680591; doi:10.3390/molecules24142632)
Supplement: Supplementary file 1 [file molecules-24-02632-s001.pdf]

# Annular tautomerism of 3(5)-disubstituted-1*H*-pyrazoles with ester and amide group

Anna Kusakiewicz-Dawid, Monika Porada, Błażej Dziuk, Dawid Siodłak*
Faculty of Chemistry, University of Opole, Oleska 48, 45-052 Opole, Poland,
e-mail: dsiodlak@uni.opole.pl

## Table of contents


| | | |
|---|---|---|
| **Table 1S.** | Selected geometric parameters for *N*-Methyl 5-methyl-1H-pyrazole-3-carboxylamide *(***1a***)* $CH_3$-Pz-CONHCH$_3$ (Å,°) | 3 |
| **Table 2S.** | Selected geometric parameters for ethyl 5-methyl-1H-pyrazole-3-carboxylate (**1b**) $CH_3$-Pz-COOCH$_2$CH$_3$ (Å, °) | 5 |
| **Table 3S.** | Selected geometric parameters for methyl 5-amino-1H-pyrazole-3-carboxylate (**2b**) $H_2$N-Pz-COOCH$_3$ (Å, °) | 6 |
| **Table 4S**. | Selected geometric parameters for methyl 3-nitro-1H-pyrazole-5-carboxylate (**3b**) $N_2$O-Pz-COOCH$_3$ (Å, °) | 9 |
| **Table 5S.** | Selected geometric parameters for Methylammonium 3-nitro-1H-pyrazole-5-carboxylate (**4**) $N_2$OPzCOO$^-$MeNH$_3^+$ (Å, °) | 10 |
| **Table 6S.** | Selected hydrogen-bond parameters | 11 |
| **Table 7S.** | Selected parameter of the 3(5) substituted pyrazole structures with carbonyl (amide/ester) as substituent | 12 |
| **Figure 1S.** | Tautomers and conformers of the studied molecules calculated *in vacuo*. | 14 |
| **Figure 2S.** | The change of relative energy (kcal/mol) for the structures (see Table 3) od the studied compounds **1-3**. | 15 |
| **Figure 3S.** | $^1$H NMR spectrum of *N*-methyl 5-methyl-1*H*-pyrazole-3-carboxylamide (**1a**) in $CDCl_3$. | 16 |
| **Figure 4S.** | $^1$H NMR spectrum of *N*-methyl 5-methyl-1*H*-pyrazole-3-carboxylamide (**1a**) in DMSO-d$_6$. | 16 |
| **Figure 5S.** | $^1$H NMR spectrum of *N*-methyl 5-methyl-1*H*-pyrazole-3-carboxylamide (**1a**) in $CD_3OD$. | 17 |
| **Figure 6S.** | NOE spectrum of *N*-methyl 5-methyl-1*H*-pyrazole-3-carboxylamide (**1a**) in DMSO-d$_6$. | 17 |
| **Figure 7S.** | NOE spectrum of *N*-methyl 5-methyl-1*H*-pyrazole-3-carboxylamide (**1a**) in DMSO-d$_6$. | 18 |
| **Figure 8S.** | $^1$H NMR spectrum of ethyl 5-methyl-1*H*-pyrazole-3-carboxylate (**1b**) in $CDCl_3$. | 18 |
| **Figure 9S.** | $^1$H NMR spectrum of ethyl 5-methyl-1*H*-pyrazole-3-carboxylate (**1b**) in DMSO-d$_6$. | 19 |
| **Figure 10S.** | $^1$H NMR spectrum of ethyl 5-methyl-1*H*-pyrazole-3-carboxylate (**1b**) in MeOD. | 19 |
| **Figure 11S.** | NOE spectrum of ethyl 5-methyl-1*H*-pyrazole-3-carboxylate (**1b**) in DMSO-d$_6$. | 20 |
| **Figure 12S.** | NOE spectrum of ethyl 5-methyl-1*H*-pyrazole-3-carboxylate (**1b**) in DMSO-d$_6$. | 20 |
| **Figure 13S.** | $^1$H NMR spectrum of methyl 5(3)-amino-1*H*-pyrazole-3(5)-carboxylate (**2b**) in $CDCl_3$. | 21 |
| **Figure 14S.** | $^1$H NMR spectrum of methyl 5(3)-amino-1*H*-pyrazole-3(5)-carboxylate (**2b**) in DMSO-d$_6$. | 21 |
| **Figure 15S.** | $^{13}$C NMR spectrum of methyl 5(3)-amino-1*H*-pyrazole-3(5)-carboxylate (**2b**) in DMSO-d$_6$. | 22 |
| **Figure 16S.** | $^1$H NMR spectrum of methyl 5(3)-amino-1*H*-pyrazole-3(5)-carboxylate (**2b**) in $CD_3OD$. | 22 |
| **Figure 17S.** | NOE spectrum of methyl 5(3)-amino-1*H*-pyrazole-3(5)-carboxylate (**2b**) in DMSO-d$_6$. | 23 |
| **Figure 18S.** | $^1$H NMR spectrum of methyl 3-nitro-1*H*-pyrazole-5-carboxylate (**3b**) in $CDCl_3$. | 23 |
| **Figure 19S.** | $^1$H NMR spectrum of methyl 3-nitro-1*H*-pyrazole-5-carboxylate (**3b**) in DMSO-d$_6$. | 24 |
| **Figure 20S.** | $^1$H NMR spectrum of methyl 3-nitro-1*H*-pyrazole-5-carboxylate (**3b**) in $CD_3OD$. | 24 |
| **Figure 21S.** | NOE spectrum of methyl 3-nitro-1*H*-pyrazole-5-carboxylate (**3b**) in DMSO-d$_6$. | 25 |
| **Figure 22S.** | DSC of ethyl 5-methyl-1*H*-pyrazole-3-carboxylate (**1b**) | 26 |
| **Figure 23S.** | DSC of methyl 5(3)-amino-1*H*-pyrazole-3(5)-carboxylate (**2b**) | 27 |
| **Figure 24S.** | DSC of methyl 3-nitro-1*H*-pyrazole-5-carboxylate (**3b**) | 28 |

**Supplementary Materials**

**Table 1S.** Selected geometric parameters for *N*-Methyl 5-methyl-1H-pyrazole-3-carboxylamide ***(1a)*** $CH_3$-Pz-CONHC$H_3$ (Å, º)

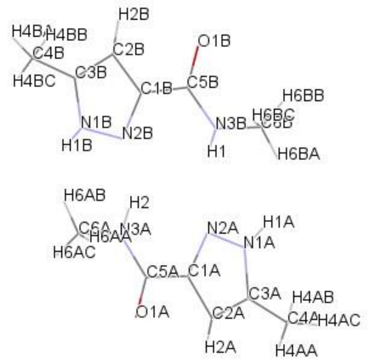


| | | | |
|---|---|---|---|
| N1A—C3A | 1.3512 (19) | C2B—C3B | 1.375 (2) |
| N1A—N2A | 1.3515 (17) | C2B—H2B | 0.9300 |
| N1A—H1A | 0.8600 | C4A—H4AA | 0.9600 |
| O1A—C5A | 1.2424 (17) | C4A—H4AB | 0.9600 |
| C1A—N2A | 1.3438 (18) | C4A—H4AC | 0.9600 |
| C1A—C2A | 1.3952 (19) | C3B—C4B | 1.490 (2) |
| C1A—C5A | 1.477 (2) | N3B—C5B | 1.3367 (19) |
| N1B—C3B | 1.3484 (19) | N3B—C6B | 1.4556 (18) |
| N1B—N2B | 1.3505 (16) | N3B—H1 | 0.8600 |
| N1B—H1B | 0.8600 | C4B—H4BA | 0.9600 |
| O1B—C5B | 1.2395 (16) | C4B—H4BB | 0.9600 |
| C1B—N2B | 1.3438 (17) | C4B—H4BC | 0.9600 |
| C1B—C2B | 1.395 (2) | C6A—H6AA | 0.9600 |
| C1B—C5B | 1.4817 (19) | C6A—H6AB | 0.9600 |
| C3A—C2A | 1.374 (2) | C6A—H6AC | 0.9600 |
| C3A—C4A | 1.494 (2) | C6B—H6BA | 0.9600 |
| N3A—C5A | 1.3287 (18) | C6B—H6BB | 0.9600 |
| N3A—C6A | 1.4510 (18) | C6B—H6BC | 0.9600 |
| N3A—H2 | 0.8600 | C2A—H2A | 0.9300 |
| C3A—N1A—N2A | 113.48 (12) | C2B—C3B—C4B | 132.29 (14) |
| C3A—N1A—H1A | 123.3 | C5B—N3B—C6B | 120.93 (12) |
| N2A—N1A—H1A | 123.3 | C5B—N3B—H1 | 119.5 |
| N2A—C1A—C2A | 111.91 (13) | C6B—N3B—H1 | 119.5 |
| N2A—C1A—C5A | 121.72 (13) | O1A—C5A—N3A | 122.62 (13) |
| C2A—C1A—C5A | 126.35 (13) | O1A—C5A—C1A | 119.47 (13) |
| C1A—N2A—N1A | 103.33 (12) | N3A—C5A—C1A | 117.90 (12) |
| C3B—N1B—N2B | 113.51 (11) | C3B—C4B—H4BA | 109.5 |
| C3B—N1B—H1B | 123.2 | C3B—C4B—H4BB | 109.5 |
| N2B—N1B—H1B | 123.2 | H4BA—C4B—H4BB | 109.5 |
| N2B—C1B—C2B | 111.55 (12) | C3B—C4B—H4BC | 109.5 |
| N2B—C1B—C5B | 121.71 (13) | H4BA—C4B—H4BC | 109.5 |
| C2B—C1B—C5B | 126.74 (13) | H4BB—C4B—H4BC | 109.5 |
| N1A—C3A—C2A | 105.99 (13) | N3A—C6A—H6AA | 109.5 |
| N1A—C3A—C4A | 121.81 (14) | N3A—C6A—H6AB | 109.5 |
| C2A—C3A—C4A | 132.19 (14) | H6AA—C6A—H6AB | 109.5 |

| | | | |
|---|---|---|---|
| C5A—N3A—C6A | 120.94 (12) | N3A—C6A—H6AC | 109.5 |
| C5A—N3A—H2 | 119.5 | H6AA—C6A—H6AC | 109.5 |
| C6A—N3A—H2 | 119.5 | H6AB—C6A—H6AC | 109.5 |
| C1B—N2B—N1B | 103.58 (11) | N3B—C6B—H6BA | 109.5 |
| C3B—C2B—C1B | 105.48 (13) | N3B—C6B—H6BB | 109.5 |
| C3B—C2B—H2B | 127.3 | H6BA—C6B—H6BB | 109.5 |
| C1B—C2B—H2B | 127.3 | N3B—C6B—H6BC | 109.5 |
| C3A—C4A—H4AA | 109.5 | H6BA—C6B—H6BC | 109.5 |
| C3A—C4A—H4AB | 109.5 | H6BB—C6B—H6BC | 109.5 |
| H4AA—C4A—H4AB | 109.5 | O1B—C5B—N3B | 123.08 (13) |
| C3A—C4A—H4AC | 109.5 | O1B—C5B—C1B | 120.03 (13) |
| H4AA—C4A—H4AC | 109.5 | N3B—C5B—C1B | 116.89 (12) |
| H4AB—C4A—H4AC | 109.5 | C3A—C2A—C1A | 105.28 (13) |
| N1B—C3B—C2B | 105.88 (12) | C3A—C2A—H2A | 127.4 |
| N1B—C3B—C4B | 121.83 (13) | C1A—C2A—H2A | 127.4 |
| C2A—C1A—N2A—N1A | 0.16 (15) | C6A—N3A—C5A—C1A | −178.40 (12) |
| C5A—C1A—N2A—N1A | −178.50 (12) | N2A—C1A—C5A—O1A | 165.29 (13) |
| C3A—N1A—N2A—C1A | −0.35 (15) | C2A—C1A—C5A—O1A | −13.2 (2) |
| N2A—N1A—C3A—C2A | 0.41 (16) | N2A—C1A—C5A—N3A | −15.4 (2) |
| N2A—N1A—C3A—C4A | 179.69 (13) | C2A—C1A—C5A—N3A | 166.12 (13) |
| C2B—C1B—N2B—N1B | −0.13 (15) | C6B—N3B—C5B—O1B | −2.0 (2) |
| C5B—C1B—N2B—N1B | 178.95 (12) | C6B—N3B—C5B—C1B | 178.53 (13) |
| C3B—N1B—N2B—C1B | −0.21 (15) | N2B—C1B—C5B—O1B | 165.12 (13) |
| N2B—C1B—C2B—C3B | 0.40 (16) | C2B—C1B—C5B—O1B | −16.0 (2) |
| C5B—C1B—C2B—C3B | −178.62 (13) | N2B—C1B—C5B—N3B | −15.4 (2) |
| N2B—N1B—C3B—C2B | 0.46 (16) | C2B—C1B—C5B—N3B | 163.51 (13) |
| N2B—N1B—C3B—C4B | −179.12 (12) | N1A—C3A—C2A—C1A | −0.28 (15) |
| C1B—C2B—C3B—N1B | −0.49 (15) | C4A—C3A—C2A—C1A | −179.45 (15) |
| C1B—C2B—C3B—C4B | 179.02 (15) | N2A—C1A—C2A—C3A | 0.08 (16) |
| C6A—N3A—C5A—O1A | 0.9 (2) | C5A—C1A—C2A—C3A | 178.66 (13) |

## Supplementary Materials

**Table 2S.** Selected geometric parameters for ethyl 5-methyl-1H-pyrazole-3-carboxylate (**1b**) $CH_3$-Pz-$COOCH_2CH_3$ (Å, º)

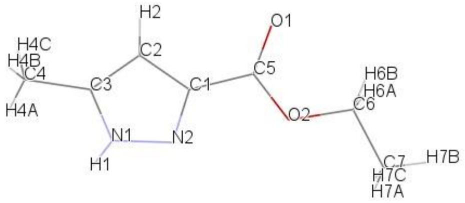


| | | | |
|---|---|---|---|
| N1—N2 | 1.3458 (17) | C3—C4 | 1.491 (2) |
| N1—C3 | 1.3512 (19) | C4—H4A | 0.9600 |
| N1—H1 | 0.8600 | C4—H4B | 0.9600 |
| N2—C1 | 1.3423 (18) | C4—H4C | 0.9600 |
| O1—C5 | 1.2087 (18) | C6—C7 | 1.501 (2) |
| O2—C5 | 1.3399 (18) | C6—H6A | 0.9700 |
| O2—C6 | 1.4578 (18) | C6—H6B | 0.9700 |
| C1—C2 | 1.400 (2) | C7—H7A | 0.9600 |
| C1—C5 | 1.475 (2) | C7—H7B | 0.9600 |
| C2—C3 | 1.377 (2) | C7—H7C | 0.9600 |
| C2—H2 | 0.9300 | | |
| N2—N1—C3 | 113.56 (12) | H4A—C4—H4C | 109.5 |
| N2—N1—H1 | 123.2 | H4B—C4—H4C | 109.5 |
| C3—N1—H1 | 123.2 | O1—C5—O2 | 124.52 (14) |
| C1—N2—N1 | 103.78 (12) | O1—C5—C1 | 123.77 (14) |
| C5—O2—C6 | 116.16 (11) | O2—C5—C1 | 111.70 (12) |
| N2—C1—C2 | 111.54 (13) | O2—C6—C7 | 107.29 (12) |
| N2—C1—C5 | 120.39 (13) | O2—C6—H6A | 110.3 |
| C2—C1—C5 | 128.06 (13) | C7—C6—H6A | 110.3 |
| C3—C2—C1 | 105.25 (13) | O2—C6—H6B | 110.3 |
| C3—C2—H2 | 127.4 | C7—C6—H6B | 110.3 |
| C1—C2—H2 | 127.4 | H6A—C6—H6B | 108.5 |
| N1—C3—C2 | 105.88 (13) | C6—C7—H7A | 109.5 |
| N1—C3—C4 | 121.39 (13) | C6—C7—H7B | 109.5 |
| C2—C3—C4 | 132.73 (14) | H7A—C7—H7B | 109.5 |
| C3—C4—H4A | 109.5 | C6—C7—H7C | 109.5 |
| C3—C4—H4B | 109.5 | H7A—C7—H7C | 109.5 |
| H4A—C4—H4B | 109.5 | H7B—C7—H7C | 109.5 |
| C3—C4—H4C | 109.5 | | |
| C3—N1—N2—C1 | −0.54 (16) | C1—C2—C3—C4 | 179.66 (16) |
| N1—N2—C1—C2 | 0.76 (16) | C6—O2—C5—O1 | −0.2 (2) |
| N1—N2—C1—C5 | −179.66 (12) | C6—O2—C5—C1 | −179.37 (12) |
| N2—C1—C2—C3 | −0.71 (17) | N2—C1—C5—O1 | 175.04 (15) |
| C5—C1—C2—C3 | 179.75 (15) | C2—C1—C5—O1 | −5.5 (3) |
| N2—N1—C3—C2 | 0.11 (17) | N2—C1—C5—O2 | −5.8 (2) |
| N2—N1—C3—C4 | −179.30 (13) | C2—C1—C5—O2 | 173.72 (14) |
| C1—C2—C3—N1 | 0.35 (16) | C5—O2—C6—C7 | 174.94 (12) |

## Supplementary Materials

**Table 3S.** Selected geometric parameters for methyl 5-amino-1H-pyrazole-3-carboxylate (**2b**) $H_2N$-Pz-$COOCH_3$ (Å, º)

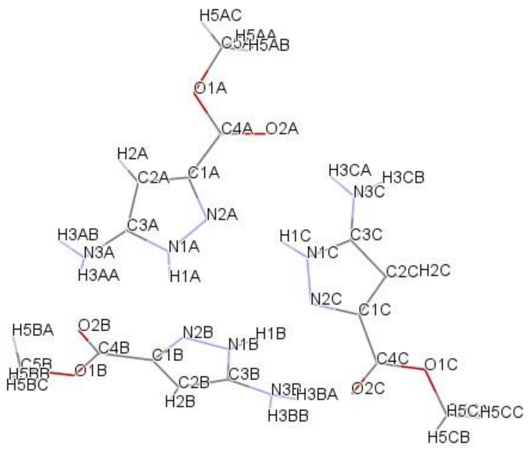


| | | | |
|---|---|---|---|
| C1C—N2C | 1.3351 (19) | N1B—C3B | 1.3503 (19) |
| C1C—C2C | 1.399 (2) | N1B—N2B | 1.3524 (17) |
| C1C—C4C | 1.475 (2) | N1B—H1B | 0.8600 |
| C2C—C3C | 1.384 (2) | O1B—C4B | 1.3386 (18) |
| C2C—H2C | 0.9300 | O1B—C5B | 1.4452 (18) |
| C3C—N1C | 1.3537 (19) | C1B—N2B | 1.3411 (19) |
| C3C—N3C | 1.370 (2) | C1B—C2B | 1.402 (2) |
| C4C—O2C | 1.2191 (18) | C1B—C4B | 1.473 (2) |
| C4C—O1C | 1.3368 (18) | N3A—C3A | 1.378 (2) |
| C5C—O1C | 1.4493 (18) | N3A—H3AB | 0.895 (17) |
| C5C—H5CA | 0.9600 | N3A—H3AA | 0.877 (18) |
| C5C—H5CB | 0.9600 | N3B—C3B | 1.387 (2) |
| C5C—H5CC | 0.9600 | N3B—H3BA | 0.892 (18) |
| N1A—C3A | 1.3562 (19) | N3B—H3BB | 0.880 (18) |
| N1A—N2A | 1.3582 (17) | C3B—C2B | 1.379 (2) |
| N1A—H1A | 0.8600 | N3C—H3CA | 0.906 (18) |
| O1A—C4A | 1.3381 (18) | N3C—H3CB | 0.856 (18) |
| O1A—C5A | 1.4459 (18) | C4B—O2B | 1.2131 (18) |
| C1A—N2A | 1.3389 (19) | C5B—H5BA | 0.9600 |
| C1A—C2A | 1.400 (2) | C5B—H5BB | 0.9600 |
| C1A—C4A | 1.476 (2) | C5B—H5BC | 0.9600 |
| O2A—C4A | 1.2139 (18) | C5A—H5AA | 0.9600 |
| C2A—C3A | 1.374 (2) | C5A—H5AB | 0.9600 |
| C2A—H2A | 0.9300 | C5A—H5AC | 0.9600 |
| N1C—N2C | 1.3629 (17) | C2B—H2B | 0.9300 |
| N1C—H1C | 0.8600 | | |
| N2C—C1C—C2C | 112.41 (14) | C2B—C1B—C4B | 129.00 (14) |
| N2C—C1C—C4C | 118.92 (13) | C3A—N3A—H3AB | 114.3 (11) |
| C2C—C1C—C4C | 128.66 (14) | C3A—N3A—H3AA | 115.6 (11) |
| C3C—C2C—C1C | 104.68 (13) | H3AB—N3A—H3AA | 115.7 (15) |
| C3C—C2C—H2C | 127.7 | N1A—C3A—C2A | 106.98 (13) |

| | | | |
|---|---|---|---|
| C1C—C2C—H2C | 127.7 | N1A—C3A—N3A | 121.33 (14) |
| N1C—C3C—N3C | 121.78 (14) | C2A—C3A—N3A | 131.52 (15) |
| N1C—C3C—C2C | 106.44 (13) | C1C—N2C—N1C | 103.77 (12) |
| N3C—C3C—C2C | 131.54 (15) | C3B—N3B—H3BA | 113.5 (11) |
| O2C—C4C—O1C | 123.72 (15) | C3B—N3B—H3BB | 113.8 (11) |
| O2C—C4C—C1C | 125.03 (14) | H3BA—N3B—H3BB | 110.2 (16) |
| O1C—C4C—C1C | 111.25 (13) | N1B—C3B—C2B | 106.73 (14) |
| O1C—C5C—H5CA | 109.5 | N1B—C3B—N3B | 120.63 (14) |
| O1C—C5C—H5CB | 109.5 | C2B—C3B—N3B | 132.55 (15) |
| H5CA—C5C—H5CB | 109.5 | C3C—N3C—H3CA | 115.4 (11) |
| O1C—C5C—H5CC | 109.5 | C3C—N3C—H3CB | 116.5 (12) |
| H5CA—C5C—H5CC | 109.5 | H3CA—N3C—H3CB | 112.8 (16) |
| H5CB—C5C—H5CC | 109.5 | O2B—C4B—O1B | 123.40 (14) |
| C3A—N1A—N2A | 112.41 (12) | O2B—C4B—C1B | 125.50 (14) |
| C3A—N1A—H1A | 123.8 | O1B—C4B—C1B | 111.09 (13) |
| N2A—N1A—H1A | 123.8 | O2A—C4A—O1A | 123.97 (14) |
| C4A—O1A—C5A | 115.06 (12) | O2A—C4A—C1A | 125.53 (14) |
| N2A—C1A—C2A | 112.38 (14) | O1A—C4A—C1A | 110.47 (13) |
| N2A—C1A—C4A | 118.68 (14) | O1B—C5B—H5BA | 109.5 |
| C2A—C1A—C4A | 128.93 (14) | O1B—C5B—H5BB | 109.5 |
| C1A—N2A—N1A | 103.74 (12) | H5BA—C5B—H5BB | 109.5 |
| C3A—C2A—C1A | 104.49 (13) | O1B—C5B—H5BC | 109.5 |
| C3A—C2A—H2A | 127.8 | H5BA—C5B—H5BC | 109.5 |
| C1A—C2A—H2A | 127.8 | H5BB—C5B—H5BC | 109.5 |
| C3C—N1C—N2C | 112.68 (13) | O1A—C5A—H5AA | 109.5 |
| C3C—N1C—H1C | 123.7 | O1A—C5A—H5AB | 109.5 |
| N2C—N1C—H1C | 123.7 | H5AA—C5A—H5AB | 109.5 |
| C4C—O1C—C5C | 115.80 (12) | O1A—C5A—H5AC | 109.5 |
| C3B—N1B—N2B | 112.86 (13) | H5AA—C5A—H5AC | 109.5 |
| C3B—N1B—H1B | 123.6 | H5AB—C5A—H5AC | 109.5 |
| N2B—N1B—H1B | 123.6 | C1B—N2B—N1B | 103.87 (12) |
| C4B—O1B—C5B | 115.12 (12) | C3B—C2B—C1B | 104.54 (14) |
| N2B—C1B—C2B | 111.97 (14) | C3B—C2B—H2B | 127.7 |
| N2B—C1B—C4B | 118.99 (13) | C1B—C2B—H2B | 127.7 |
| N2C—C1C—C2C—C3C | 0.21 (17) | C3C—N1C—N2C—C1C | −1.09 (16) |
| C4C—C1C—C2C—C3C | −178.98 (15) | N2B—N1B—C3B—C2B | 1.16 (17) |
| C1C—C2C—C3C—N1C | −0.85 (16) | N2B—N1B—C3B—N3B | 178.19 (13) |
| C1C—C2C—C3C—N3C | 173.41 (16) | C5B—O1B—C4B—O2B | 0.6 (2) |
| N2C—C1C—C4C—O2C | 3.8 (2) | C5B—O1B—C4B—C1B | −178.37 (12) |
| C2C—C1C—C4C—O2C | −177.06 (15) | N2B—C1B—C4B—O2B | −3.9 (2) |
| N2C—C1C—C4C—O1C | −175.76 (13) | C2B—C1B—C4B—O2B | 178.40 (15) |
| C2C—C1C—C4C—O1C | 3.4 (2) | N2B—C1B—C4B—O1B | 175.00 (13) |
| C2A—C1A—N2A—N1A | −0.87 (16) | C2B—C1B—C4B—O1B | −2.7 (2) |
| C4A—C1A—N2A—N1A | −179.72 (12) | C5A—O1A—C4A—O2A | 1.0 (2) |
| C3A—N1A—N2A—C1A | 0.51 (15) | C5A—O1A—C4A—C1A | −177.21 (12) |
| N2A—C1A—C2A—C3A | 0.90 (17) | N2A—C1A—C4A—O2A | −13.4 (2) |
| C4A—C1A—C2A—C3A | 179.60 (14) | C2A—C1A—C4A—O2A | 167.97 (15) |

## Supplementary Materials

| | | | |
|---|---|---|---|
| N3C—C3C—N1C—N2C | −173.69 (13) | N2A—C1A—C4A—O1A | 164.82 (12) |
| C2C—C3C—N1C—N2C | 1.25 (16) | C2A—C1A—C4A—O1A | −13.8 (2) |
| O2C—C4C—O1C—C5C | 2.4 (2) | C2B—C1B—N2B—N1B | 0.84 (16) |
| C1C—C4C—O1C—C5C | −177.99 (12) | C4B—C1B—N2B—N1B | −177.20 (12) |
| N2A—N1A—C3A—C2A | 0.03 (16) | C3B—N1B—N2B—C1B | −1.24 (16) |
| N2A—N1A—C3A—N3A | −175.67 (13) | N1B—C3B—C2B—C1B | −0.57 (16) |
| C1A—C2A—C3A—N1A | −0.53 (16) | N3B—C3B—C2B—C1B | −177.10 (16) |
| C1A—C2A—C3A—N3A | 174.56 (16) | N2B—C1B—C2B—C3B | −0.18 (17) |
| C2C—C1C—N2C—N1C | 0.51 (16) | C4B—C1B—C2B—C3B | 177.61 (14) |
| C4C—C1C—N2C—N1C | 179.78 (13) | | |

**Supplementary Materials**

**Table 4S**. Selected geometric parameters for methyl 3-nitro-1H-pyrazole-5-carboxylate (**3b**) $N_2O$-Pz-$COOCH_3$ (Å, º)

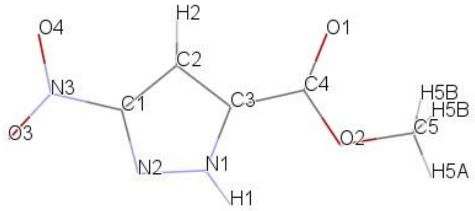


| | | | |
|---|---|---|---|
| O1—C4 | 1.2135 (19) | O2—C5 | 1.464 (2) |
| N1—N2 | 1.3363 (19) | C2—C3 | 1.376 (2) |
| N1—C3 | 1.360 (2) | C2—H2 | 0.9300 |
| N1—H1 | 0.8600 | O4—N3 | 1.2279 (18) |
| C1—N2 | 1.328 (2) | C4—C3 | 1.479 (2) |
| C1—C2 | 1.393 (2) | O3—N3 | 1.2233 (18) |
| C1—N3 | 1.453 (2) | C5—H5B | 0.997 (15) |
| O2—C4 | 1.3226 (19) | C5—H5A | 0.98 (2) |
| N2—N1—C3 | 112.57 (12) | O1—C4—C3 | 123.30 (14) |
| N2—N1—H1 | 123.7 | O2—C4—C3 | 111.55 (13) |
| C3—N1—H1 | 123.7 | N1—C3—C2 | 107.37 (14) |
| N2—C1—C2 | 114.17 (14) | N1—C3—C4 | 122.70 (14) |
| N2—C1—N3 | 119.22 (14) | C2—C3—C4 | 129.93 (14) |
| C2—C1—N3 | 126.61 (15) | O3—N3—O4 | 125.44 (14) |
| C1—N2—N1 | 103.21 (12) | O3—N3—C1 | 118.22 (14) |
| C4—O2—C5 | 115.28 (12) | O4—N3—C1 | 116.34 (14) |
| C3—C2—C1 | 102.68 (13) | O2—C5—H5B | 109.0 (8) |
| C3—C2—H2 | 128.7 | O2—C5—H5A | 104.4 (12) |
| C1—C2—H2 | 128.7 | H5B—C5—H5A | 112.3 (10) |
| O1—C4—O2 | 125.15 (15) | | |
| C2—C1—N2—N1 | 0.000 (1) | C1—C2—C3—C4 | 180.000 (1) |
| N3—C1—N2—N1 | 180.000 (1) | O1—C4—C3—N1 | 180.000 (1) |
| C3—N1—N2—C1 | 0.000 (1) | O2—C4—C3—N1 | 0.000 (1) |
| N2—C1—C2—C3 | 0.000 (1) | O1—C4—C3—C2 | 0.000 (1) |
| N3—C1—C2—C3 | 180.000 (1) | O2—C4—C3—C2 | 180.000 (1) |
| C5—O2—C4—O1 | 0.000 (1) | N2—C1—N3—O3 | 0.000 (1) |
| C5—O2—C4—C3 | 180.000 (1) | C2—C1—N3—O3 | 180.000 (1) |
| N2—N1—C3—C2 | 0.000 (1) | N2—C1—N3—O4 | 180.000 (1) |
| N2—N1—C3—C4 | 180.000 (1) | C2—C1—N3—O4 | 0.000 (1) |
| C1—C2—C3—N1 | 0.000 (1) | | |

**Supplementary Materials**

**Table 5S.** Selected geometric parameters for Methylammonium 3-nitro-1H-pyrazole-5-carboxylate (**4**) $N_2OPzCOO^-MeNH_3^+$ (Å, º)

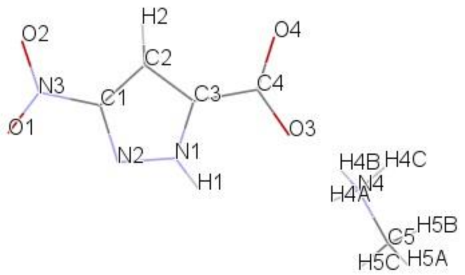


| | | | |
|---|---|---|---|
| O1—N3 | 1.2314 (13) | C3—C4 | 1.4981 (18) |
| C1—N2 | 1.3309 (16) | O3—C4 | 1.2502 (16) |
| C1—C2 | 1.3902 (18) | C4—O4 | 1.2537 (15) |
| C1—N3 | 1.4441 (16) | N4—C5 | 1.4811 (16) |
| N1—N2 | 1.3379 (15) | N4—H4A | 0.8900 |
| N1—C3 | 1.3568 (16) | N4—H4B | 0.8900 |
| N1—H1 | 0.8600 | N4—H4C | 0.8900 |
| O2—N3 | 1.2247 (14) | C5—H5A | 0.9600 |
| C2—C3 | 1.3766 (18) | C5—H5B | 0.9600 |
| C2—H2 | 0.9300 | C5—H5C | 0.9600 |
| N2—C1—C2 | 114.22 (11) | O3—C4—O4 | 126.23 (12) |
| N2—C1—N3 | 118.07 (11) | O3—C4—C3 | 114.88 (11) |
| C2—C1—N3 | 127.70 (12) | O4—C4—C3 | 118.88 (12) |
| N2—N1—C3 | 113.31 (11) | C5—N4—H4A | 109.5 |
| N2—N1—H1 | 123.3 | C5—N4—H4B | 109.5 |
| C3—N1—H1 | 123.3 | H4A—N4—H4B | 109.5 |
| C3—C2—C1 | 103.08 (12) | C5—N4—H4C | 109.5 |
| C3—C2—H2 | 128.5 | H4A—N4—H4C | 109.5 |
| C1—C2—H2 | 128.5 | H4B—N4—H4C | 109.5 |
| C1—N2—N1 | 102.63 (10) | N4—C5—H5A | 109.5 |
| N1—C3—C2 | 106.76 (11) | N4—C5—H5B | 109.5 |
| N1—C3—C4 | 119.15 (11) | H5A—C5—H5B | 109.5 |
| C2—C3—C4 | 134.08 (12) | N4—C5—H5C | 109.5 |
| O2—N3—O1 | 124.14 (11) | H5A—C5—H5C | 109.5 |
| O2—N3—C1 | 117.43 (11) | H5B—C5—H5C | 109.5 |
| O1—N3—C1 | 118.43 (11) | | |
| N2—C1—C2—C3 | 0.23 (15) | N2—C1—N3—O2 | 172.97 (11) |
| N3—C1—C2—C3 | −178.55 (13) | C2—C1—N3—O2 | −8.3 (2) |
| C2—C1—N2—N1 | 0.17 (14) | N2—C1—N3—O1 | −7.09 (18) |
| N3—C1—N2—N1 | 179.07 (11) | C2—C1—N3—O1 | 171.64 (13) |
| C3—N1—N2—C1 | −0.53 (14) | N1—C3—C4—O3 | −0.02 (18) |
| N2—N1—C3—C2 | 0.70 (15) | C2—C3—C4—O3 | −178.86 (14) |
| N2—N1—C3—C4 | −178.43 (11) | N1—C3—C4—O4 | 179.45 (12) |
| C1—C2—C3—N1 | −0.53 (14) | C2—C3—C4—O4 | 0.6 (2) |
| C1—C2—C3—C4 | 178.42 (14) | | |

## Supplementary Materials

**Table 6S.** Selected hydrogen-bond parameters

| *D*—H···*A* | *D*—H (Å) | H···*A* (Å) | *D*···*A* (Å) | *D*—H···*A* (°) |
|---|---|---|---|---|
| **1a** | | | | |
| N1A—H1A···O1A$^{i}$ | 0.86 | 1.90 | 2.7454 (15) | 166.9 |
| N1B—H1B···O1B$^{ii}$ | 0.86 | 1.95 | 2.7815 (15) | 163.1 |
| N3A—H2···N2B | 0.86 | 2.20 | 3.0254 (17) | 159.6 |
| C4A—H4AC···O1A$^{iii}$ | 0.96 | 2.51 | 3.2622 (19) | 135.1 |
| N3B—H1···N2A | 0.86 | 2.22 | 3.0532 (17) | 164.1 |
| C2A—H2A···N2A$^{iv}$ | 0.93 | 2.65 | 3.5627 (19) | 166.0 |
| **1b** | | | | |
| C4—H4C···O1$^{v}$ | 0.96 | 2.66 | 3.5453 (19) | 153.9 |
| N1—H1···N2$^{vi}$ | 0.86 | 2.17 | 2.9852 (18) | 157.2 |
| N1—H1···O2$^{vi}$ | 0.86 | 2.48 | 3.0998 (16) | 129.3 |
| **2b** | | | | |
| C5C—H5CA···O2A$^{vii}$ | 0.96 | 2.60 | 3.255 (2) | 125.4 |
| N1A—H1A···N2B | 0.86 | 2.05 | 2.8559 (18) | 155.2 |
| N1C—H1C···N2A | 0.86 | 2.06 | 2.8957 (18) | 165.1 |
| N1B—H1B···N2C | 0.86 | 2.00 | 2.8519 (18) | 169.5 |
| N3A—H3AB···O2C$^{viii}$ | 0.895 (17) | 2.361 (18) | 3.1325 (18) | 144.4 (14) |
| N3A—H3AA···O2B | 0.877 (18) | 2.057 (19) | 2.9198 (19) | 167.5 (16) |
| N3B—H3BA···O2C | 0.892 (18) | 2.126 (19) | 3.0023 (19) | 166.8 (16) |
| N3C—H3CA···O2A | 0.906 (18) | 2.059 (19) | 2.9602 (19) | 173.0 (16) |
| N3C—H3CB···N3B$^{ix}$ | 0.856 (18) | 2.495 (18) | 3.203 (2) | 140.6 (15) |
| C5A—H5AB···N3A$^{viii}$ | 0.96 | 2.65 | 3.511 (2) | 150.1 |
| **3b** | | | | |
| N1—H1···O1$^{x}$ | 0.86 | 2.01 | 2.8274 (17) | 159.0 |
| C2—H2···N2$^{xi}$ | 0.93 | 2.35 | 3.250 (2) | 163.6 |
| C5—H5A···O4$^{xii}$ | 0.98 (2) | 2.63 (2) | 3.563 (2) | 158.9 (16) |
| **4** | | | | |
| N1—H1···O3$^{xiii}$ | 0.86 | 2.54 | 3.0467 (14) | 118.5 |
| N1—H1···O4$^{xiii}$ | 0.86 | 2.08 | 2.9340 (15) | 175.1 |
| C2—H2···O2$^{xiv}$ | 0.93 | 2.53 | 3.3703 (16) | 151.2 |
| N4—H4A···O4$^{xv}$ | 0.89 | 1.96 | 2.8146 (15) | 160.6 |
| N4—H4B···O3 | 0.89 | 1.85 | 2.7299 (14) | 167.8 |
| N4—H4C···N2$^{xvi}$ | 0.89 | 2.22 | 3.0874 (16) | 166.3 |

Symmetry code(s): (i) *x*, −*y*+1/2, *z*+1/2; (ii) *x*, *y*−1, *z*; (iii) −*x*+1, *y*+1/2, −*z*+1/2; (iv) −*x*+1, *y*−1/2, −*z*+1/2; (v) −*x*+1/2, *y*+1/2, −*z*+1/2; (vi) −*x*+3/2, *y*+1/2, −*z*+1/2; (vii) −*x*+1/2, *y*+1/2, −*z*+3/2; (viii) −*x*+3/2, *y*−1/2, −*z*+3/2; (ix) −*x*+1/2, *y*−1/2, −*z*+3/2; (x) *x*−1, *y*, *z*; (xi) *x*+1, *y*, *z*; (xii) *x*, *y*, *z*+1; (xiii) *x*−1/2, −*y*+3/2, *z*−1/2; (xiv) −*x*+1, −*y*+1, −*z*+1; (xv) *x*+1/2, −*y*+3/2, *z*−1/2; (xvi) *x*+3/2, −*y*+3/2, *z*+1/2.

## Supplementary Materials

**Table 7S.** Selected parameter of the 3(5) substituted pyrazole structures with carbonyl (amide/ester) as substituent

| Refcode | Tautomer | | Substituent position | | Conformer | | | References | Compound name |
|---|---|---|---|---|---|---|---|---|---|
| | T3 | T5 | C-3 | C-5 | cis | trans | | | |
| ANIKIT | 3 | | Me- | CONHN- | -9.22 | | T3c | K.Karrouchi, Y.Ramli, J.Taoufik, S.Radi, M.Ansar, J.T.Mague Unknown (2016), 1, x160793 | (Z)-5-Methyl-N'-(4-methylbenzylidene)-1H-pyrazole-3-carbohydrazide |
| EZIYES | 3 | | Me- | CONHN- | -9.30 | | T3c | S.K.Seth, N.C.Saha, S.Ghosh, T.Kar Chem.Phys.Lett. (2011), 506, 309 | 5-Methyl-1H-pyrazole-3-carbohydrazide monohydrate |
| RISHEH | 3 | | Me- | CONHCH2 | -11.78 | | T3c | Jing Hu, Guofeng Chen, Guochun Ma, Hongguang Song Acta Crystallogr.,Sect.E:Struct.Rep.Online (2007), 63, o4806 | 5,5'-Dimethyl-N,N'-(iminodiethylene)di-1H-pyrazole-3-carboxamide |
| VEYQIB | 3 | | Me- | CONHN- | 3.24 | | T3c | S.Konar, A.Jana, K.Das, S.Ray, S.Chatterjee, S.K.Kar S.Konar, A.Jana, K.Das, S.Ray, S.Chatterjee, S.K.Kar | Methyl (((5-methyl-1H-pyrazol-3-yl)carbonyl)hydrazono)(phenyl)acetate |
| VUXQEM | 3 | | Me- | CONHN- | -5.50 | | T3c | K.Karrouchi, M.Ansar, S.Radi, M.Saadi, L.El Ammari Acta Cryst. E (2015), 71, o890 | N'-(Diphenylmethylene)-5-methyl-1H-pyrazole-3-carbohydrazide |
| FAQSAR | 3 | | Me- | COOEt | 5.45 | | T3c | L.Infantes, C.Foces-Foces, R.M.Claramunt, C.Lopez, N.Jagerovic, J.Elguero Heterocycles (1999), 50, 227 | 3-Ethoxycarbonyl-5-methylpyrazole |
| | | | | | | | | | |
| MUXMIC | 3 | | H- | CONHPh | 9.40 | | T3c | M.Kissane, S.E.Lawrence, A.R.Maguire M.Kissane, S.E.Lawrence, A.R.Maguire | N-(4-Methylphenyl)-1H-pyrazole-3-carboxamide |
| NIJCOZ | 3 | | H- | CONHtBu | 1.88 | | T3c | R.M.Claramunt, M.A.Garcia, C.Lopez, S.Trofimenko, G.P.A.Yap, I.Alkorta, J.Elguero Magn.Reson.Chem. (2005), 43, 89 | 1H-Pyrazole-3-(N-t-butyl)carboxamide |
| FAQROE | 3 | | H- | COOEt | | 179.40 | T3t | L.Infantes, C.Foces-Foces, R.M.Claramunt, C.Lopez, N.Jagerovic, J.Elguero Heterocycles (1999), 50, 227 | 3-Ethoxycarbonylpyrazole |
| RUNJIV | 3 | | H- | CONH2 | | -167.29 | T3t | T.T.Talele, F.R.Fronczek Private Communication (2015), , | 1H-pyrazole-3-carboxamide |
| RUNJER | 3 | | H- | COOMe | | -178.99 | T3t | T.T.Talele, F.R.Fronczek Private Communication (2015), , | methyl 1H-pyrazole-3-carboxylate |
| | | | | | | | | | |
| AFEQOS | 3 | | Ph- | COOH | -8.95 | | T3c | Xiao-Yuan Zhang, Wei Liu, Wei Tang, Yue-Bo Lai Acta Crystallogr.,Sect.E:Struct.Rep.Online (2007), 63, o3764 | 5-Phenyl-1H-pyrazole-3-carboxylic acid |
| EMUNEH | 3 | | Ph- | CONHN- | -7.27 | | T3c | K.Karrouchi, Y.Ramli, J.Taoufik, S.Radi, M.Ansar, J.T.Mague Unknown (2016), 1, x160830 | N'-[(1E)-(4-bromophenyl)methylidene]-5-phenyl-1H-pyrazole-3-carbohydrazide |
| FAQSIZ | 3 | | Ph- | COOEt | 0.96 | | T3c | L.Infantes, C.Foces-Foces, R.M.Claramunt, C.Lopez, N.Jagerovic, J.Elguero Heterocycles (1999), 50, 227 | 3-Ethoxycarbonyl-5-phenylpyrazole |
| | | | | | | | | | |
| LEPRII | 3 | | Ph- | CONHN- | -29.63 | | T3c | Fang-Fang Jian, Tong-Ling liang, Ying-Qi Qin, Huan-Qing Yu, Hai-Lian Xiao Acta Crystallogr.,Sect.E:Struct.Rep.Online (2006), 62, o4364 | 2'-(4-Methylbenzylidene)-5-phenyl-1H-pyrazole-3-carbohydrazide |
| MIRYUI | 3 | | Ph- | CONHN- | 0.19 | | T3c | Yongqi Qin, Fangfang Jian, Hailian Xiao, Jing Zhang Acta Crystallogr.,Sect.E:Struct.Rep.Online (2008), 64, o320 | (E)-5-Phenyl-N'-(1-phenylethylidene)-1H-pyrazole-3-carbohydrazide |
| QAJKUK | 3 | | Ph- | CONHN- | -12.14 | | T3c | K.Karrouchi, S.Radi, M.Ansar, J.Taoufik, H.A.Ghabbour, Y.N.Mabkhot Z.Kristallogr.-New Cryst.Struct. (2016), 231, | N'-(4-Nitrobenzylidene)-5-phenyl-1H-pyrazole-3-carbohydrazide |
| SEZDUX | 3 | | Ph- | CONHBz | -7.88 | | T3c | Yan-Qing Ge, Yong Xia, Fang Wei, Wen-Liang Dong, Bao-Xiang Zhao Acta Crystallogr.,Sect.E:Struct.Rep.Online (2007), 63, o1186 | N-Benzyl-5-phenyl-1H-pyrazole-3-carboxamide |
| UJODII | 3 | | Ph- | CONHN- | 21.58 | | T3c | K.Karrouchi, S.Radi, M.Ansar, J.Taoufik, H.A.Ghabbour, Y.N.Mabkhot Z.Kristallogr.-New Cryst.Struct. (2016), 231, | N'-(4-(Dimethylamino)benzylidene)-5-phenyl-1H-pyrazole-3-carbohydrazide |
| HUPDAY | 3 | | 4-Me-OPh- | CONHN- | 12.43 | | T3c | Yue An, Pingping Mu, Zhiping Guo, Yongheng Xing, Guanghua Zhou, Jun Ning, Jianzhou Lu, Jiehan Hu Youji Huaxue(Chin.)(Chin.J.Org.Chem.) (2009), 29, 730 | N'-(4-Methoxybenzylidene)-5-(4-methoxyphenyl)-1H-pyrazole-3-carbohydrazide |
| LAQNOI | 3 | | 4-Me-OPh- | CONH- | -2.50 | | T3c | Guangqian Han, Jiaguo Lv, Ju Zhu, Youjun Zhou, Defeng Wu Acta Crystallogr.,Sect.E:Struct.Rep.Online (2012), 68, o901 | N-(2-Hydroxyethyl)-5-(4-methoxyphenyl)-1H-pyrazole-3-carboxamide |

| | | | | | | | | | |
|---|---|---|---|---|---|---|---|---|---|
| VACYUW | 3 | | 4-Me-OPh- | CONHN- | 28.07 | | T3c | K.Karrouchi, S.Radi, M.Ansar, J.Taoufik, H.A.Ghabbour, Y.N.Mabkhot Z.Kristallogr.-New Cryst.Struct. (2016), 231, | N'-(4-Methoxybenzylidene)-5-phenyl-1H-pyrazole-3-carbohydrazide |
| GINPOJ | 3 | | Ph- | COOMe | 3.94 | | T3c | Guanghua Zhou, Yue An, Jing Han, Maofa Ge, Yongheng Xing Acta Crystallogr.,Sect.E:Struct.Rep.Online (2007), 63, o4474 | Methyl 5-phenyl-1H-pyrazole-3-carboxylate |
| RIXVOK | 3 | | 4-Me-Ph- | COOEt | -11.37 | | T3c | T.T.Dang, T.T.Dang, C.Fischer, H.Gorls, P.Langer Tetrahedron (2008), 64, 2207 | Ethyl 5-(4-tolyl)-1H-pyrazole-3-carboxylate |
| TORTOL | 3 | | 4-F-Ph- | COOEt | 7.28 | | T3c | V.Markovic, M.D.Joksovic Green Chemistry (2015), 17, 842 | Ethyl 5-(4-fluorophenyl)-1H-pyrazole-3-carboxylate |
| FAQSIZ | 3 | | Ph- | COOEt | | -156.83 | T3t | L.Infantes, C.Foces-Foces, R.M.Claramunt, C.Lopez, N.Jagerovic, J.Elguero Heterocycles (1999), 50, 227 | 3-Ethoxycarbonyl-5-phenylpyrazole |
| LOBKEU | | 5 | 4-Me-OPh- | COOEt | 11.57 | | T5c | Nana Shao, Tong Chen, Taotao Zhang, Huajian Zhu, Qunxiong Zheng, Hongbin Zou Tetrahedron (2014), 70, 795 | Ethyl 3-(4-methoxyphenyl)-1H-pyrazole-5-carboxylate |
| SEZNAN | | 5 | 2-OH-5-Me-Ph- | COOEt | 0.78 | | T5c | Yan-Qing Ge, Wen-Liang Dong, Yong Xia, Fang Wei, Bao-Xiang Zhao Acta Crystallogr.,Sect.E:Struct.Rep.Online (2007), 63, o1313 | Ethyl 3-(2-hydroxy-5-methylphenyl)-1H-pyrazole-5-carboxylate |
| VOJZEB | | 5 | 4-Br-Ph- | CONHN- | -4.16 | | T5c | Juan Sun, Peng-Cheng Lv, Yong Yin, Rong-Ju Yuan, Jian Ma, Hai-Liang Zhu Unknown (2013), 8, e69751 | 3-(4-bromophenyl)-N'-(2-chlorobenzoyl)-1H-pyrazole-5-carbohydrazide |
| HUPDEC | | 5 | 4-MeO-Ph- | CONHN- | | -155.04 | T5t | Yue An, Pingping Mu, Zhiping Guo, Yongheng Xing, Guanghua Zhou, Jun Ning, Jianzhou Lu, Jiehan Hu Youji Huaxue(Chin.)(Chin.J.Org.Chem.) (2009), 29, 730 | N'-(4-Hydroxy-3-methoxybenzylidene)-3-(4-methoxyphenyl)-1H-pyrazole-5-carbohydrazide |
| UKUVUS | | 5 | Ph- | COC=C | | 162.16 | T5t | V.V.Khlturina, Yu.V.Shklyaev, Z.A.Aliev, A.N.Maslivets Zh.Org.Khim.(Russ.)(Russ.J.Org.Chem.) (2009), 45, 1535 | 2-(8,8-Dimethyl-2,3,8,9-tetrahydro[1,4]dioxino[2,3-g]isoquinolin-6(7H)-ylidene)-1-(3-phenyl-1H-pyrazol-5-yl)ethanone |
| | | | | | | | | | |
| EWAHUH | 3 | | COO- | COOH | -7.40 | | T3c | S.Packiaraj, A.Pushpaveni, S.Govindarajan, J.M.Rawson CrystEngComm (2016), 18, 7978 | guanidinium 3-carboxy-1H-pyrazole-5-carboxylate monohydrate |
| LIGKIX | 3 | | COO- | COOH | 3.99 | | T3c | O.Ugono, N.P.Rath, A.M.Beatty CrystEngComm (2011), 13, 753 | N,N,N',N'-Tetramethylethane-1,2-diaminium bis(3-carboxy-1H-pyrazole-5-carboxylate) |
| LIGKOD | 3 | | COO- | COOH | -5.58 | | T3c | O.Ugono, N.P.Rath, A.M.Beatty CrystEngComm (2011), 13, 753 | 1,4-bis(2-Hydroxyethyl)piperazinediium bis(3-carboxy-1H-pyrazole-5-carboxylate) |
| LIGKUJ | 3 | | COO- | COOH | -4.45 | | T3c | O.Ugono, N.P.Rath, A.M.Beatty CrystEngComm (2011), 13, 753 | 2-Isopropylanilinium 3-carboxy-1H-pyrazole-5-carboxylate |
| LIGLIY | 3 | | COO- | COOH | 0.61 | | T3c | O.Ugono, N.P.Rath, A.M.Beatty CrystEngComm (2011), 13, 753 | 2-Ethoxyanilinium 3-carboxy-1H-pyrazole-5-carboxylate |
| MUPDOR | 3 | | COO- | COOH | -6.57 | | T3c | O.Ugono, N.P.Rath, A.M.Beatty Cryst.Growth Des. (2009), 9, 4595 | 2-Phenoxyanilinium 3-carboxy-1H-pyrazole-5-carboxylate |
| QUCRUC | 3 | | COO- | COOH | 13.84 | | T3c | O.Ugono, N.P.Rath, A.M.Beatty Cryst.Growth Des. (2009), 9, 4595 | 2-(4-Methoxyphenyl)ethanaminium 3-carboxy-1H-pyrazole-5-carboxylate |
| QUCSAJ | 3 | | COO- | COOH | -16.02 | | T3c | O.Ugono, N.P.Rath, A.M.Beatty Cryst.Growth Des. (2009), 9, 4595 | 2-(4-Methylphenyl)ethanaminium 3-carboxy-1H-pyrazole-5-carboxylate |
| QUCSEN | 3 | | COO- | COOH | 18.77 | | T3c | O.Ugono, N.P.Rath, A.M.Beatty Cryst.Growth Des. (2009), 9, 4595 | 2-(4-Fluorophenyl)ethanaminium 3-carboxy-1H-pyrazole-5-carboxylate |
| QUCSIR | 3 | | COO- | COOH | 10.23 | | T3c | O.Ugono, N.P.Rath, A.M.Beatty Cryst.Growth Des. (2009), 9, 4595 | 1-(1-Naphthyl)ethanaminium 3-carboxy-1H-pyrazole-5-carboxylate |
| MZCPYZ10 | 3 | | COO- | COOMe | -6.34 | | T3c | R.L.Harlow, S.H.Simonsen Acta Crystallogr.,Sect.B:Struct.Crystallogr.Cryst.Chem. (1974), 30, 2505 | 1,1,1-Trimethylhydrazinium 3-carbomethoxy-5-pyrazolecarboxylate |
| LIGKET | 3 | | COO- | COOH | | 178.42 | T3t | O.Ugono, N.P.Rath, A.M.Beatty CrystEngComm (2011), 13, 753 | Hexane-1,6-diaminium bis(3-carboxy-1H-pyrazole-5-carboxylate) |
| LIGLAQ | 3 | | COO- | COOH | | -175.29 | T3t | O.Ugono, N.P.Rath, A.M.Beatty CrystEngComm (2011), 13, 753 | 2,6-Diethylanilinium 3-carboxy-1H-pyrazole-5-carboxylate monohydrate |
| LIGLEU | 3 | | COO- | COOH | | -162.73 | T3t | O.Ugono, N.P.Rath, A.M.Beatty CrystEngComm (2011), 13, 753 | 2-Ethylanilinium 3-carboxy-1H-pyrazole-5-carboxylate monohydrate |
| MARVIM | 3 | | COO- | COOH | | 176.84 | T3t | Xiu-Ying Li, Ling Liu, Shan-Shan Wang, Miao-Miao Wang, Guang-Bo Che Z.Kristallogr.-New Cryst.Struct. (2012), 227, 73 | 4,4'-bipyridinium bis(3-carboxy-1H-pyrazole-5-carboxylate) tetrahydrate |
| PEVQUD | 3 | | COO- | COOH | | 179.14 | T3t | V.S.S.Kumar, T.Premkumar, N.P.Rath, S.Govindarajan Indian J.Chem.,Sect.B:Org.Chem.Incl.Med.Chem. (2007), 46, 141 | Hydrazonium bis(3-carboxypyrazol-5-carboxylate) |
| KUZQOM | | 5 | COO- | COOH | -3.18 | | T5c | Xin-Hui Zhou Acta Crystallogr.,Sect.E:Struct.Rep.Online (2010), 66, o2578 | Piperazine-1,4-diium bis(2-carboxy-1H-pyrazole-4-carboxylate) tetrahydrate |

| | | | | | | | | | |
|---|---|---|---|---|---|---|---|---|---|
| OFUZIY | | 5 | COO- | COOH | | -179.15 | T5t | A.M.Beatty, C.M.Schneider, A.E.Simpson, J.L.Zaher CrystEngComm (2002), 4, 282 | Propane-1,3-diammonium bis(5-carboxypyrazole-3-carboxylate) trihydrate |
| OFUZOE | | 5 | COO- | COOH | | 179.14 | T5t | A.M.Beatty, C.M.Schneider, A.E.Simpson, J.L.Zaher CrystEngComm (2002), 4, 282 | Butane-1,4-diammonium bis(5-carboxypyrazole-3-carboxylate) tetrahydrate |
| MUNZIG | | 5 | CN | COOiPro | 0.74 | | T5c | P.K.Mykhailiuk Eur.J.Org.Chem. (2015), , 7235 | Isopropyl 3-cyano-1H-pyrazole-5-carboxylate |
| MUNZOM | | 5 | CN | COCH2Ph | | 179.36 | T5t | P.K.Mykhailiuk Eur.J.Org.Chem. (2015), , 7235 | 5-(Phenylacetyl)-1H-pyrazole-3-carbonitrile |
| MUNZUS | | 5 | CN | COcBu | | 177.74 | T5t | P.K.Mykhailiuk Eur.J.Org.Chem. (2015), , 7235 | 5-(Cyclobutylcarbonyl)-1H-pyrazole-3-carbonitrile |
| MUPBAC | | 5 | CN | COCH2Bzl | | -174.71 | T5t | P.K.Mykhailiuk Eur.J.Org.Chem. (2015), , 7235 | 5-(3-Phenylpropanoyl)-1H-pyrazole-3-carbonitrile |
| APUROU | | 5 | CF3CHF | COOMe | -1.03 | | T5c | P.Mykhailiuk Private Communication (2016), , | methyl 3-(1,2,2,2-tetrafluoroethyl)-1H-pyrazole-5-carboxylate |
| APURUA | | 5 | CF3CHF | COOtBu | -4.94 | | T5c | P.Mykhailiuk Private Communication (2016), , | t-butyl 3-(1,2,2,2-tetrafluoroethyl)-1H-pyrazole-5-carboxylate |
| GOWLEL | | 5 | CHF2 | COOMe | 0.00 | | T5c | P.Mykhailiuk Angew.Chem.,Int.Ed. (2015), 54, 6558 | methyl 3-(difluoromethyl)-1H-pyrazole-5-carboxylate |
| GOWLOV | | 5 | CHF2 | COOEt | 1.30 | | T5c | P.Mykhailiuk Angew.Chem.,Int.Ed. (2015), 54, 6558 | ethyl 3-(difluoromethyl)-1H-pyrazole-5-carboxylate |
| KORWIZ | | 5 | CHF2CF2 | COOiPro | -10.46 | | T5c | P.K.Mykhailiuk Org.Biomol.Chem. (2015), 13, 3438 | isopropyl 3-(1,1,2,2-tetrafluoroethyl)-1H-pyrazole-5-carboxylate |
| WIXLUM | | 5 | CF3 | COOMe | 0.00 | | T5c | Evgeniy Y.Slobodyanyuk, O.S.Artamonov, O.V.Shishkin, P.K.Mykhailiuk Eur.J.Org.Chem. (2014), , 2487 | methyl 3-(trifluoromethyl)-1H-pyrazole-5-carboxylate |
| VOVXIP | | 5 | CHF2 | COMe | | 176.94 | T5t | P.Mykhailiuk Angew.Chem.,Int.Ed. (2015), 54, 6558 | 1-(3-(Difluoromethyl)-1H-pyrazol-5-yl)ethanone |
| APUSAH | | 5 | CF3CHF | CONMe2 | | 153.61 | T5t | P.Mykhailiuk Private Communication (2016), , | N,N-dimethyl-3-(1,2,2,2-tetrafluoroethyl)-1H-pyrazole-5-carboxamide |
| VOVXIP | | 5 | CHF2 | COMe | | 176.94 | T5t | Angew.Chem.,Int.Ed. (2015), 54, 6558 Angew.Chem.,Int.Ed. (2015), 54, 6558 | 1-(3-(Difluoromethyl)-1H-pyrazol-5-yl)ethanone |
| QARVOU | | 5 | HO- | COOEt | | 174.26 | T5t | L.Infantes, C.Foces-Foces, R.M.Claramunt, C.Lopez, J.Elguero J.Mol.Struct. (1998), 447, 71 | 5-Ethoxycarbonyl-3-hydroxypyrazole |
| | | | | | | | | | |
| APUSEL | 3 | | difluorovinyl | COOMe | 0.00 | | T3c | P.Mykhailiuk Private Communication (2016) | methyl 5-(1,2-difluorovinyl)-1H-pyrazole-3-carboxylate dihydrate |
| BIYJIE | 3 | | pherrocenyl | COOMe | -16.33 | | T3c | C.Obuah, A.Munyaneza, I.A.Guzei, J.Darkwa Dalton Trans. (2014), 43, 8940 | 3-(Ethoxycarbonyl)-5-ferrocenylpyrazole |
| VIXPAV | 3 | | Py | CONHN- | 12.22 | | T3c | S.Bala, A.Goswami, Satirtha Sengupta, Sumi Ganguly, S.Bhattacharya, S.Khanra, R.Mondal S.Bala, A.Goswami, Satirtha Sengupta, Sumi Ganguly, S.Bhattacharya, S.Khanra, R.Mondal | 5-(Pyridin-2-yl)-N'-(pyridin-2-ylmethylene)-1H-pyrazole-3-carbohydrazide acetonitrile solvate |
| ICEQAK | 3 | | CONHPh | COOEt | | 175.88 | T3t | Jing-Jing Qi, You-Jun Zhou, Xue-Fei Liu, Li-Li Ding, Can-Hui Zheng, Chun-Quan Sheng, Jia-Guo Lv, Ju Zhu Jiegou Huaxue(Chin.)(Chinese J.Struct.Chem.) (2011), 30, 1604 | Ethyl 5-(4-((phenylacetyl)amino)phenyl)-1H-pyrazole-3-carboxylate |
| PUJLIR | | 5 | 4-NO2PhCO- | COOEt | | -178.37 | T5t | Wen-Ming Shu, Jun-Rui Ma, Kai-Lu Zheng, Hui-Ying Sun, Mei Wang, Yan Yang, An-Xin Wu Tetrahedron (2014), 70, 9321 | ethyl 3-(4-nitrobenzoyl)-1H-pyrazole-5-carboxylate |
| PUJLIR | | 5 | 4-NO2PhCO- | COOEt | | -178.37 | T5t | Wen-Ming Shu, Jun-Rui Ma, Kai-Lu Zheng, Hui-Ying Sun, Mei Wang, Yan Yang, An-Xin Wu Tetrahedron (2014), 70, 9321 | ethyl 3-(4-nitrobenzoyl)-1H-pyrazole-5-carboxylate |
| YOTHIZ | | 5 | NHCSNH | COOMe | | -171.40 | T5t | B.Huang, P.-P.Kung, A.L.Rheingold, A.DiPasquale, A.Yanovsky Acta Crystallogr.,Sect.E:Struct.Rep.Online (2009), 65, o1249 | Methyl 3-(3-(ethoxycarbonyl)thioureido)-1H-pyrazole-5-carboxylate |

**Supplementary Materials**

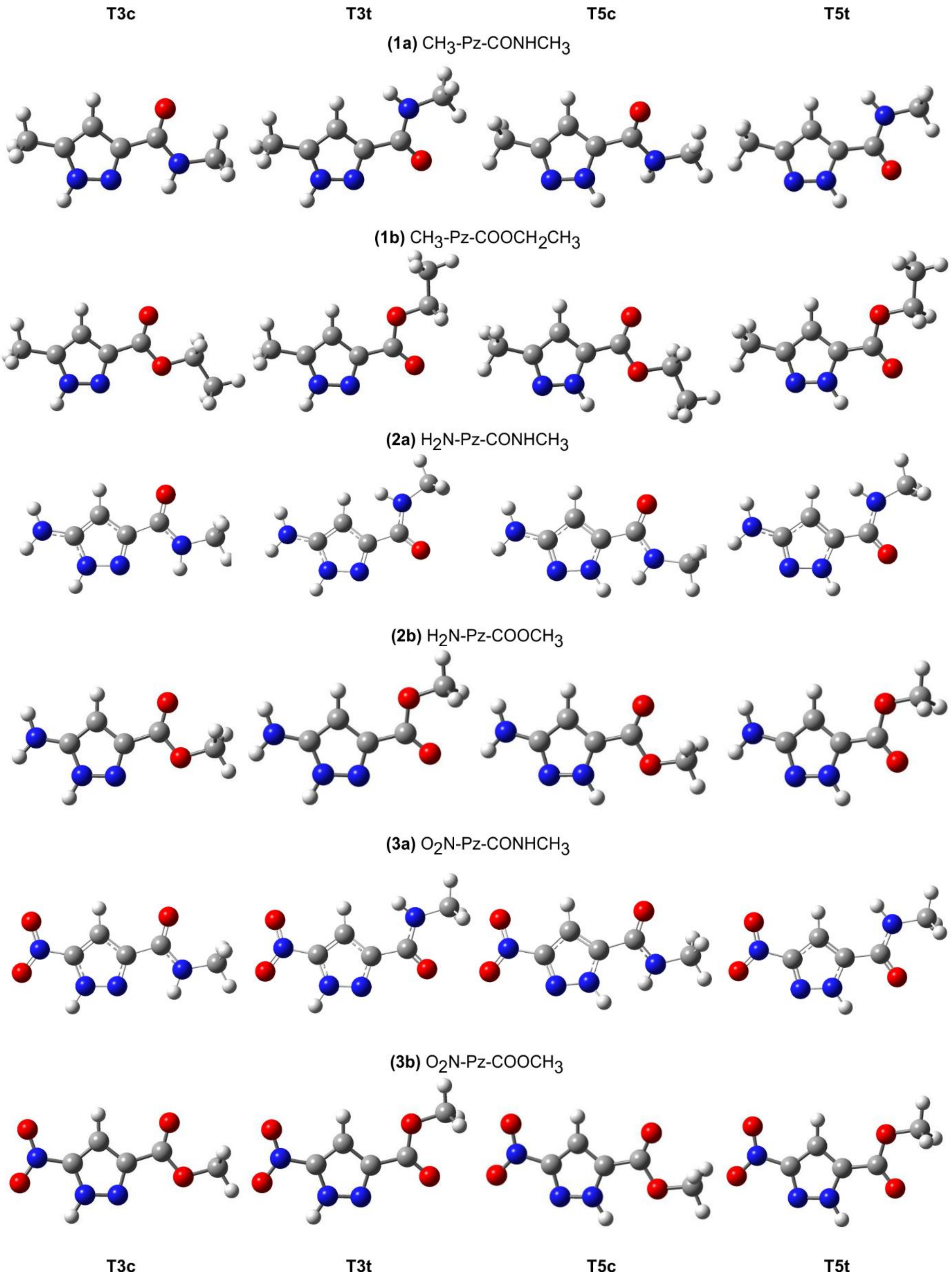


**Figure 1S.** Tautomers and conformers of the studied molecules calculated *in vacuo*.

**Supplementary Materials**

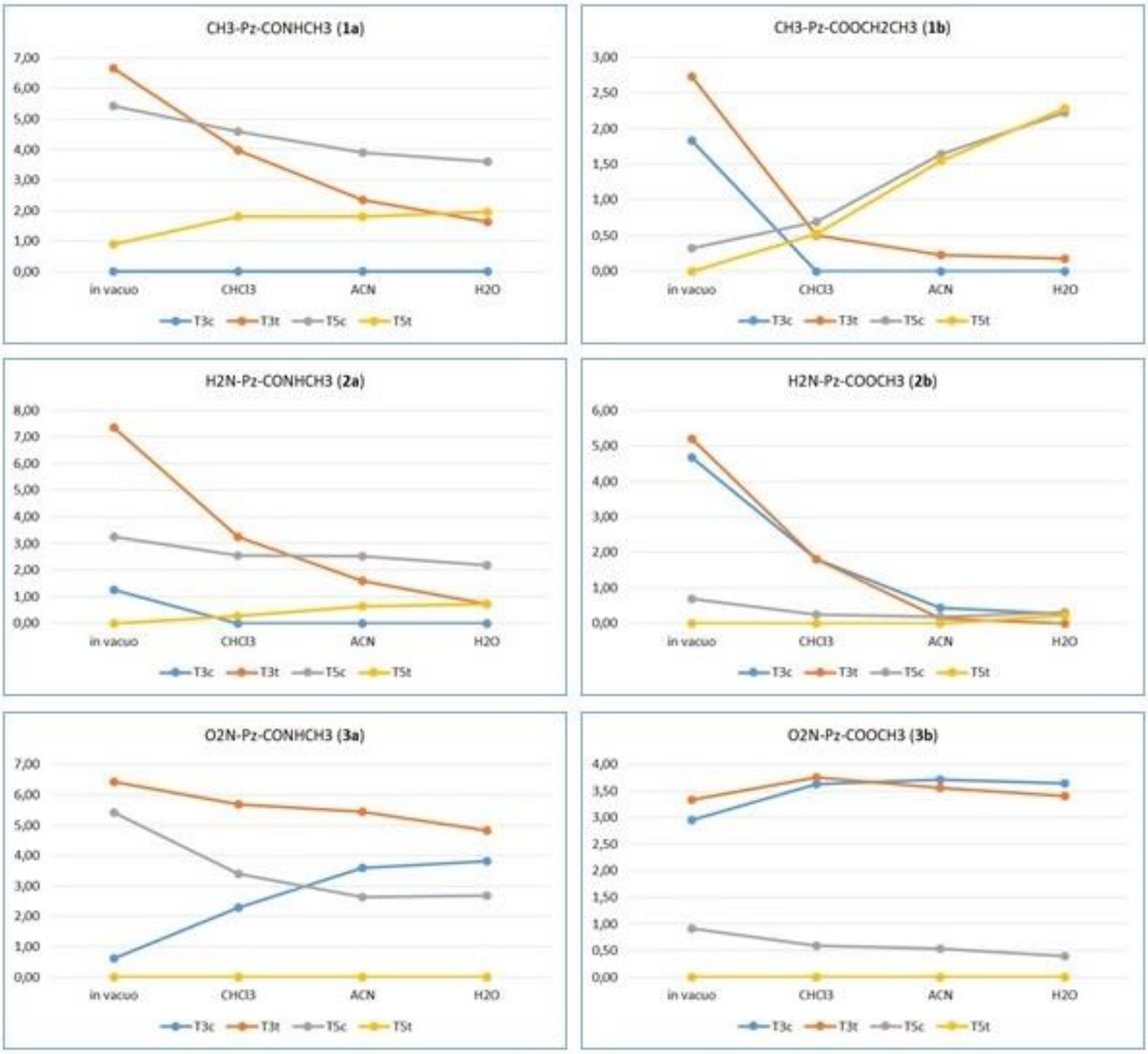


**Figure 2S.** The change of relative energy (kcal/mol) for the structures (see Table 3) od the studied compounds **1-3**.

**Supplementary Materials**

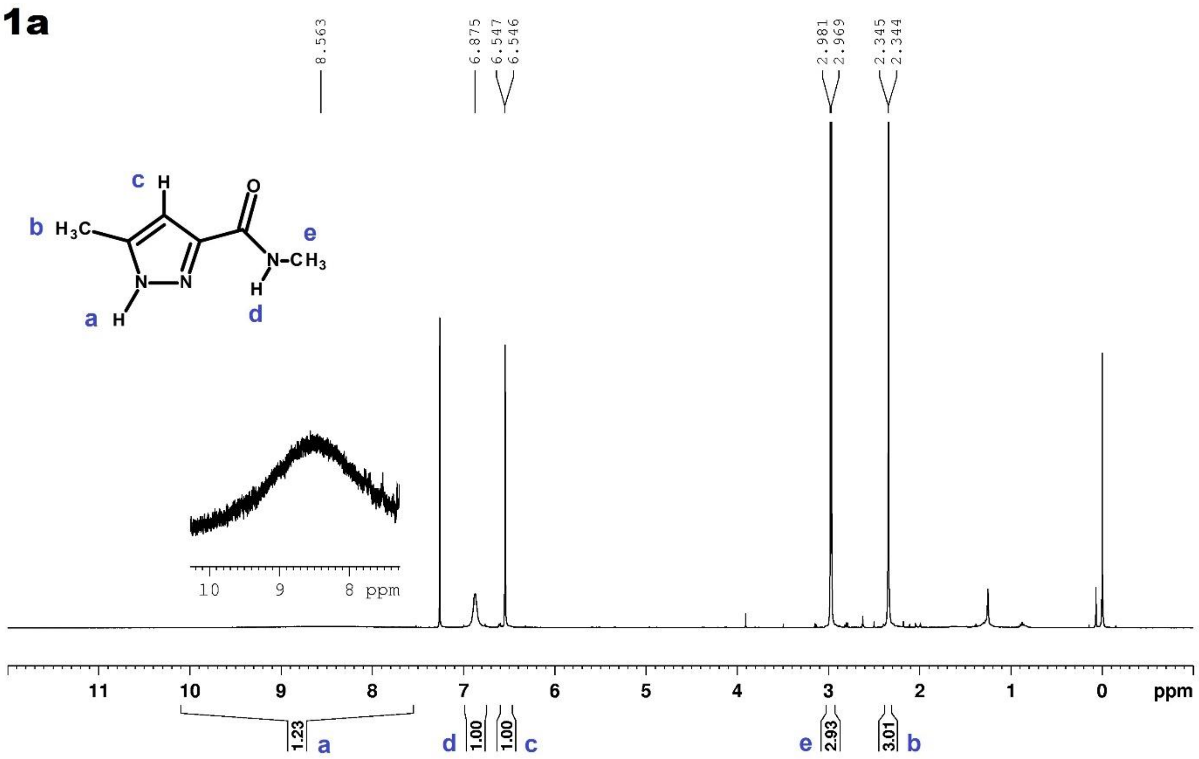


**Figure 3S.** $^{1}$H NMR spectrum of *N*-methyl 5-methyl-1*H*-pyrazole-3-carboxylamide (**1a**) in $CDCl_3$.

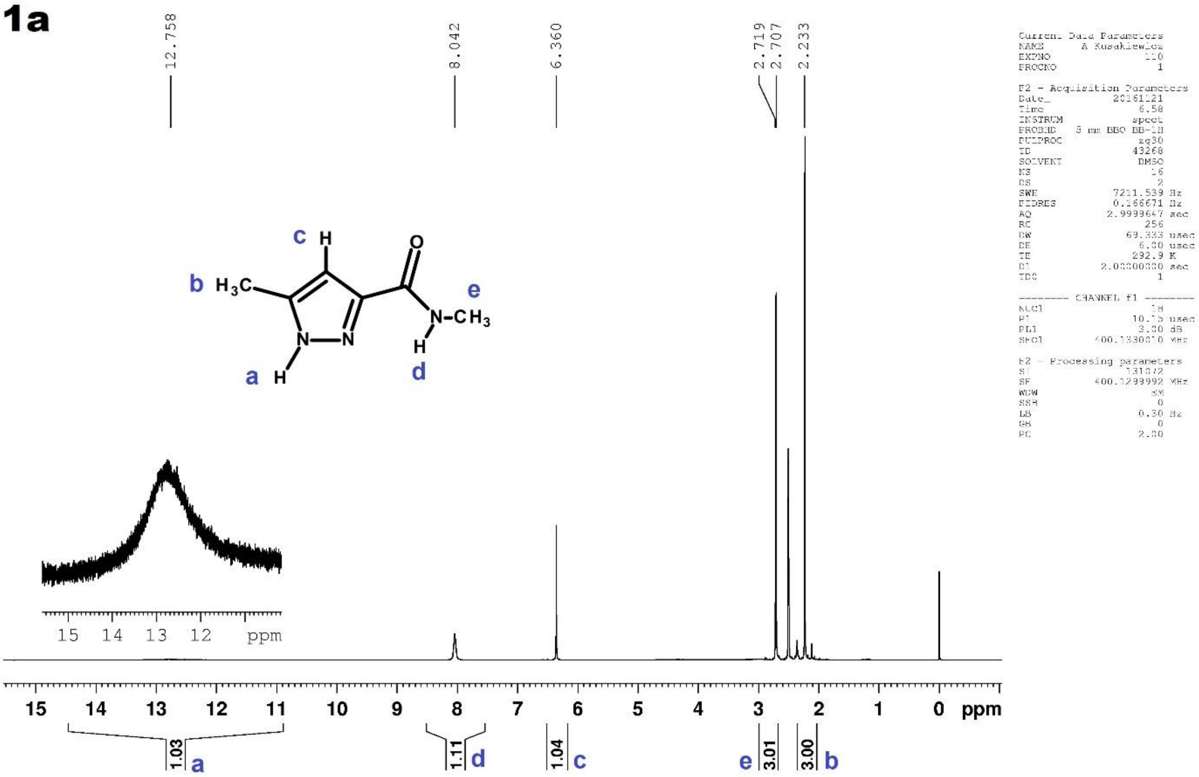


**Figure 4S.** $^{1}$H NMR spectrum of *N*-methyl 5-methyl-1*H*-pyrazole-3-carboxylamide (**1a**) in DMSO-$d_6$.

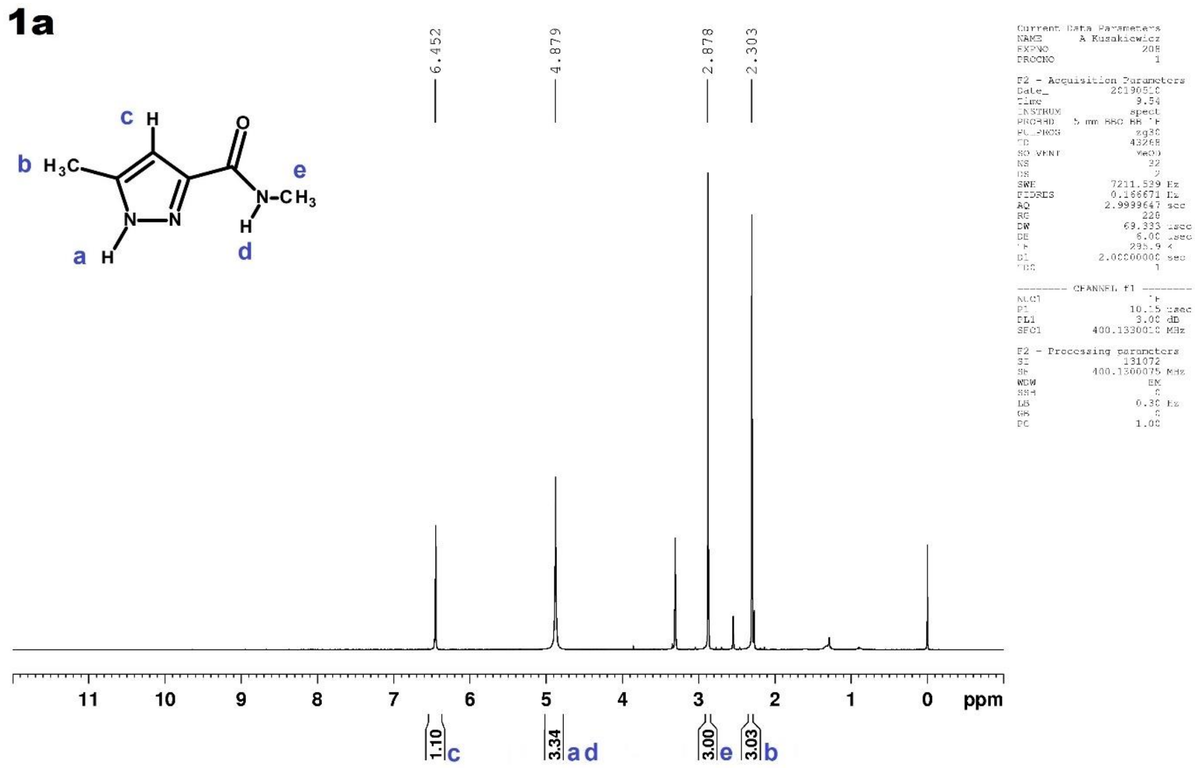


**Figure 5S.** $^{1}$H NMR spectrum of *N*-methyl 5-methyl-1*H*-pyrazole-3-carboxylamide (**1a**) in $CD_3OD$.

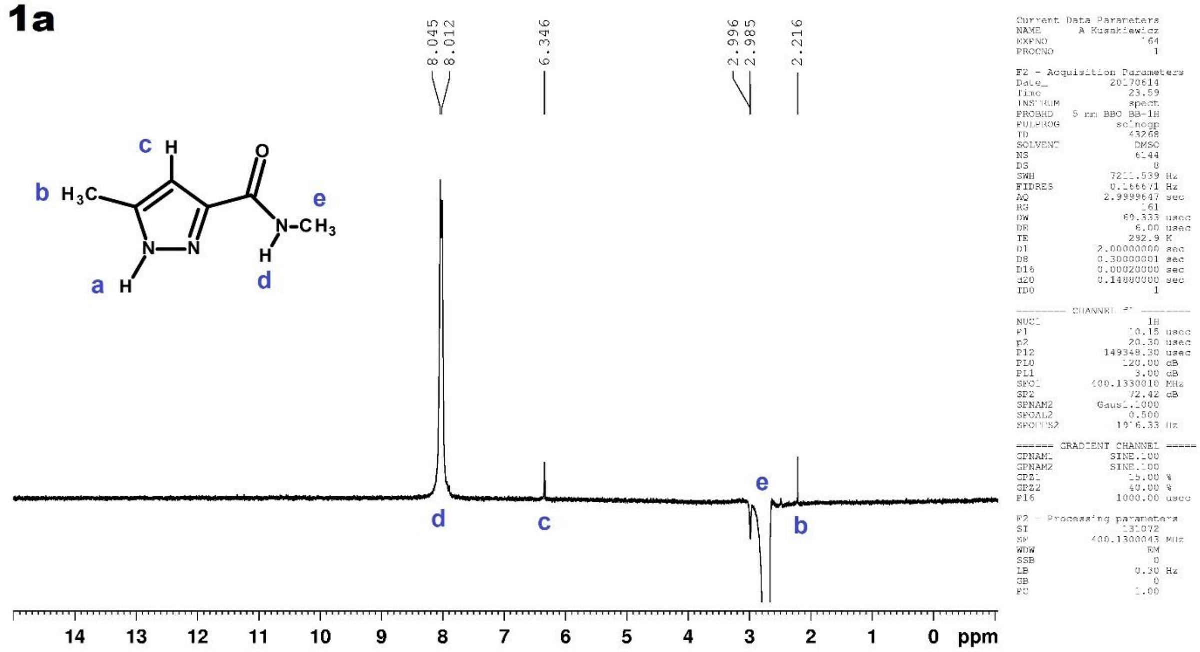


**Figure 6S.** NOE spectrum of *N*-methyl 5-methyl-1*H*-pyrazole-3-carboxylamide (**1a**) in DMSO-$d_6$.

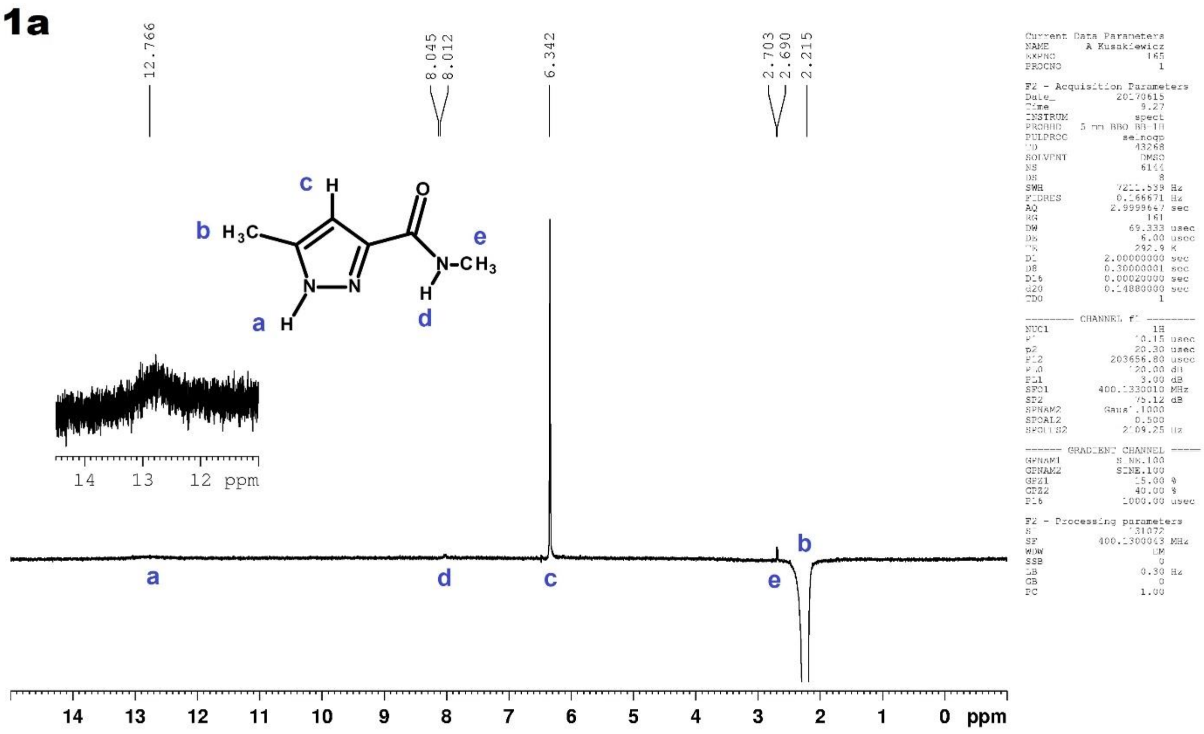


**Figure 7S.** NOE spectrum of *N*-methyl 5-methyl-*1H*-pyrazole-3-carboxylamide (**1a**) in DMSO-$d_6$.

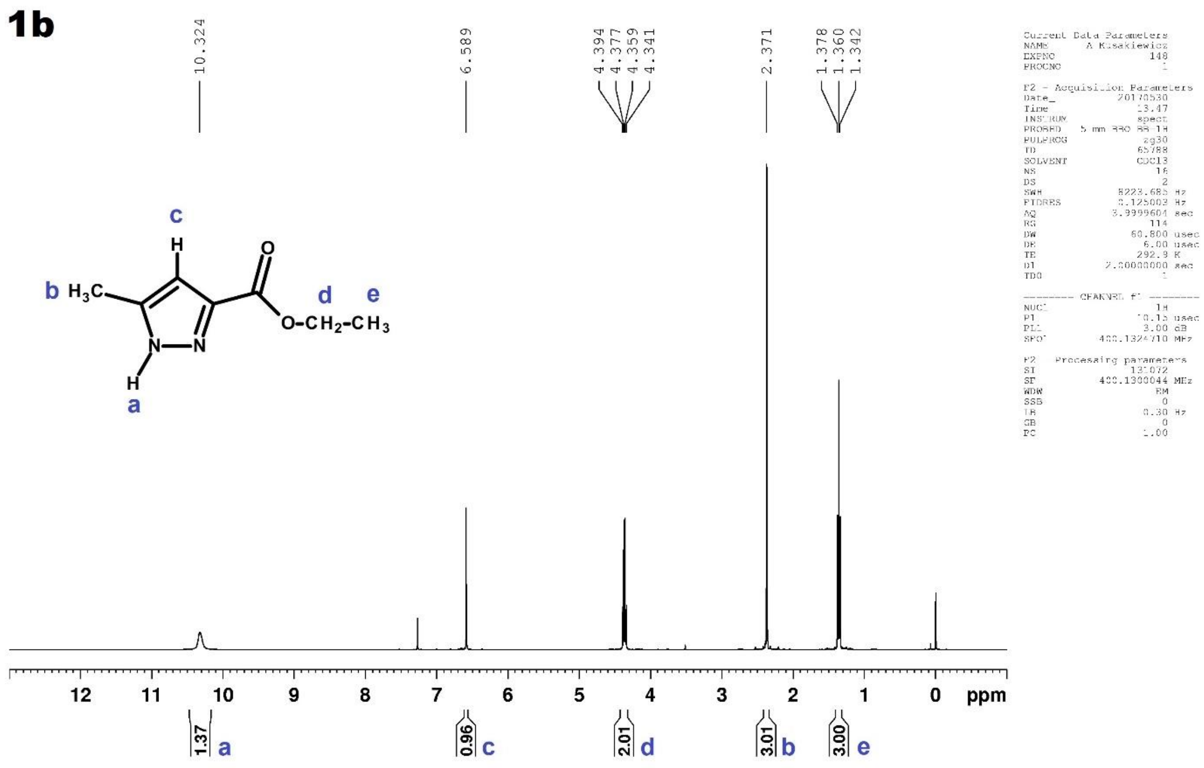


**Figure 8S.** $^{1}$H NMR spectrum of ethyl 5-methyl-1*H*-pyrazole-3-carboxylate (**1b**) in $CDCl_3$.

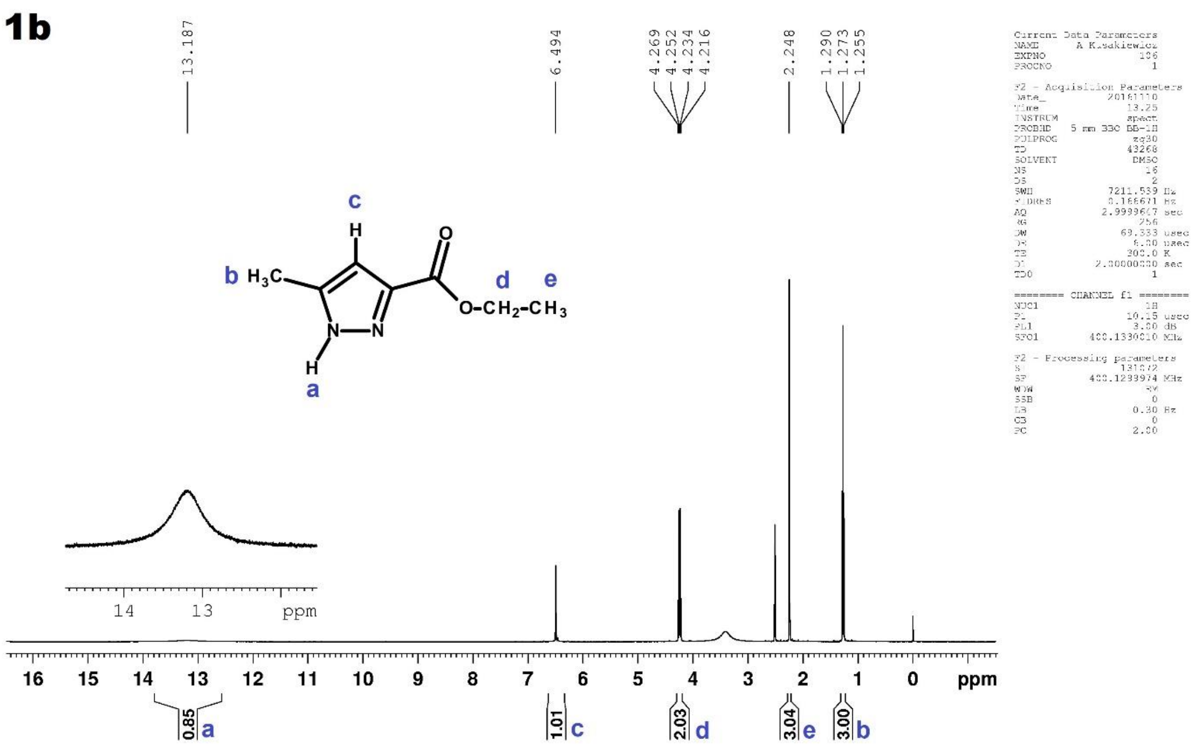


**Figure 9S.** $^{1}$H NMR spectrum of ethyl 5-methyl-1*H*-pyrazole-3-carboxylate (**1b**) in DMSO-$d_6$.

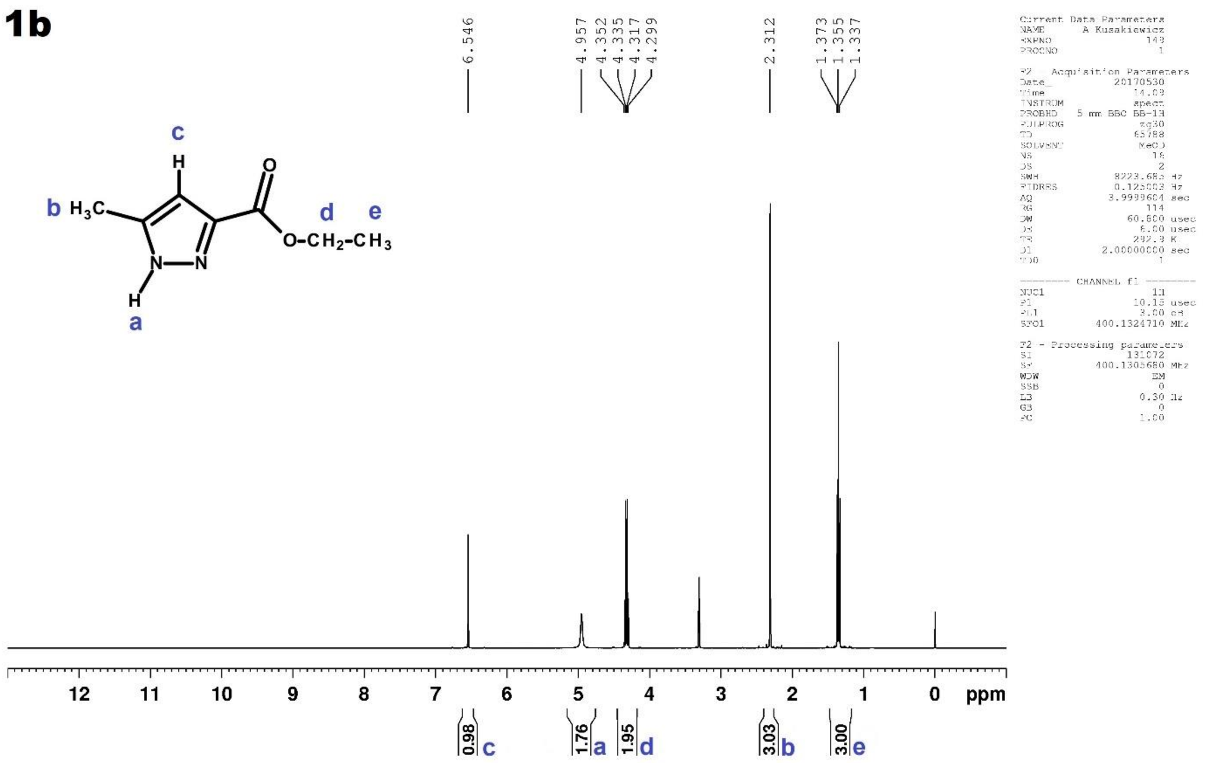


**Figure 10S.** $^{1}$H NMR spectrum of ethyl 5-methyl-1*H*-pyrazole-3-carboxylate (**1b**) in $CD_3OD$.

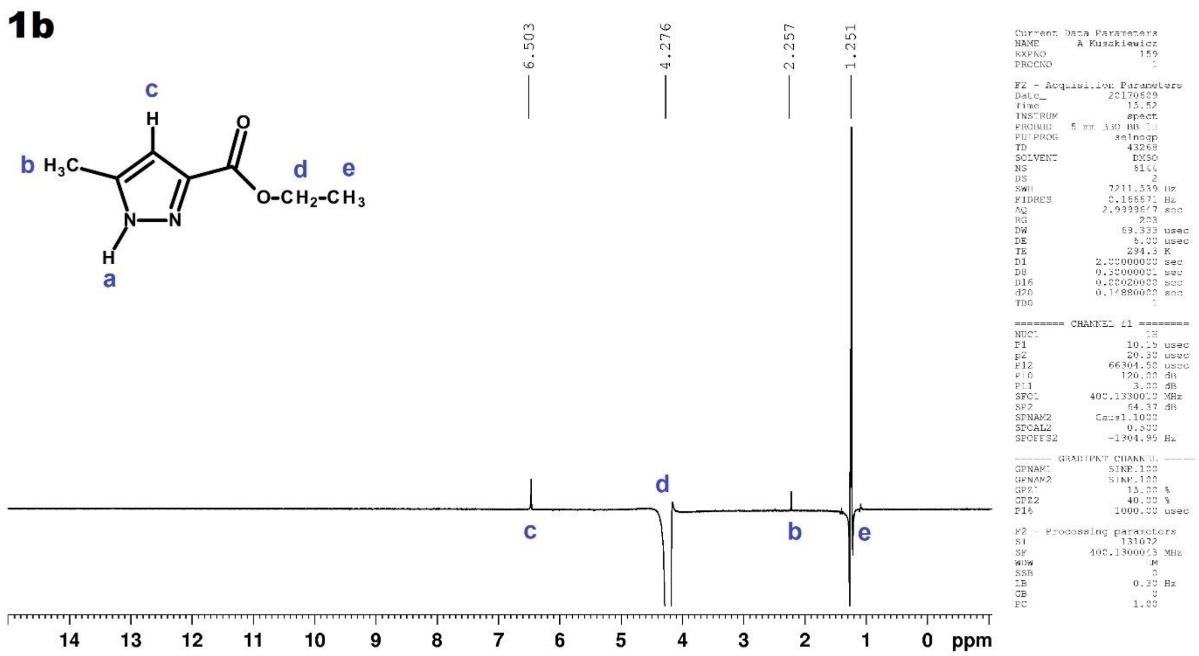


**Figure 11S.** NOE spectrum of ethyl 5-methyl-1*H*-pyrazole-3-carboxylate (**1b**) in DMSO-$d_6$.

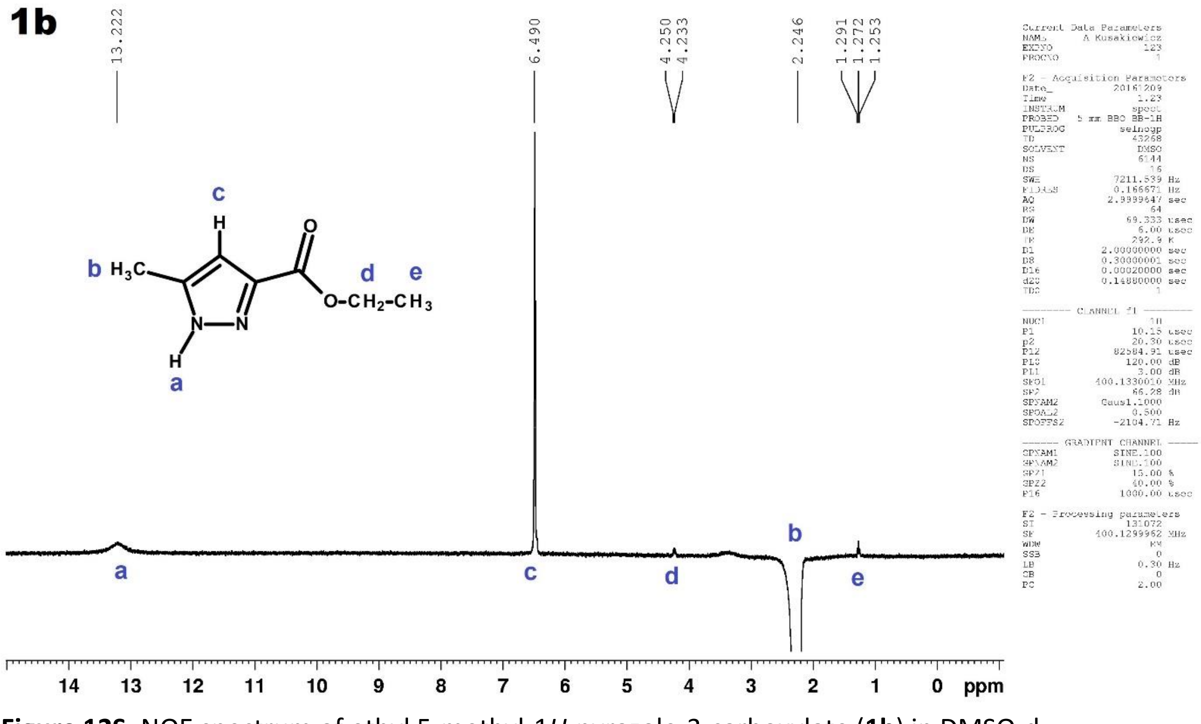


**Figure 12S.** NOE spectrum of ethyl 5-methyl-1*H*-pyrazole-3-carboxylate (**1b**) in DMSO-$d_6$.

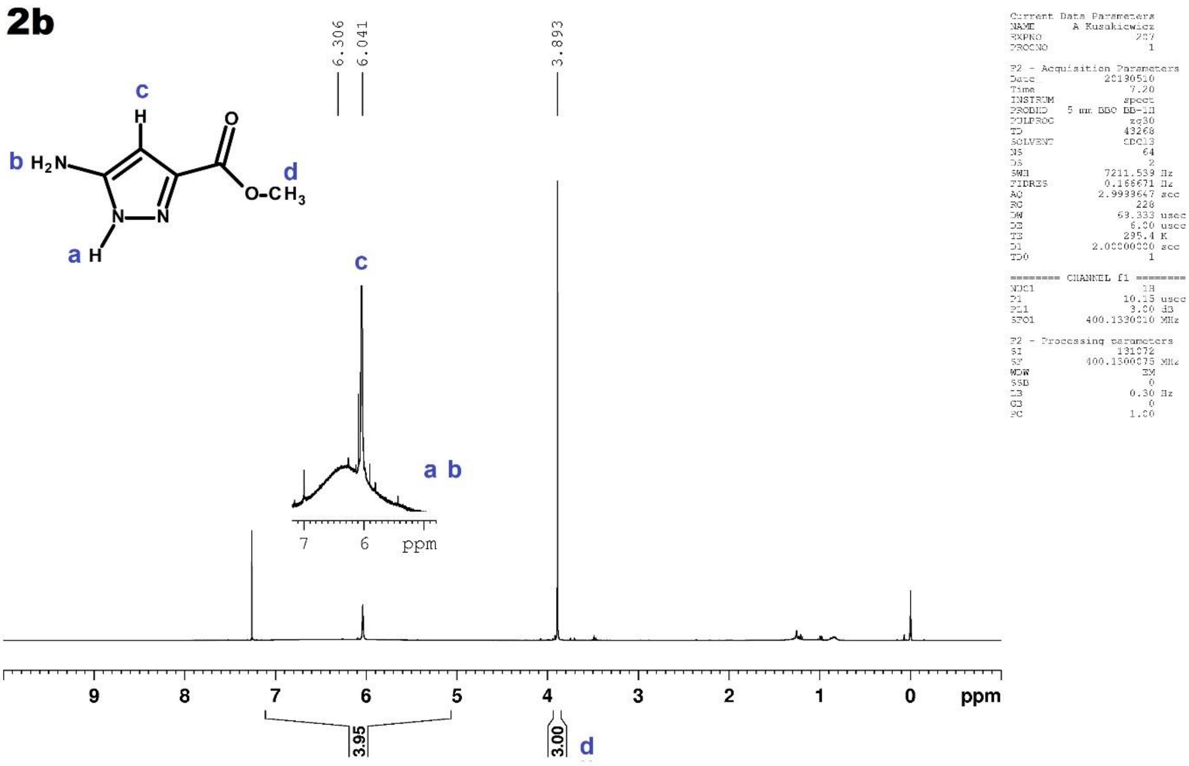


**Figure 13S.** $^{1}H$ NMR spectrum of methyl 5(3)-amino-1*H*-pyrazole-3(5)-carboxylate (**2b**) in $CDCl_3$.

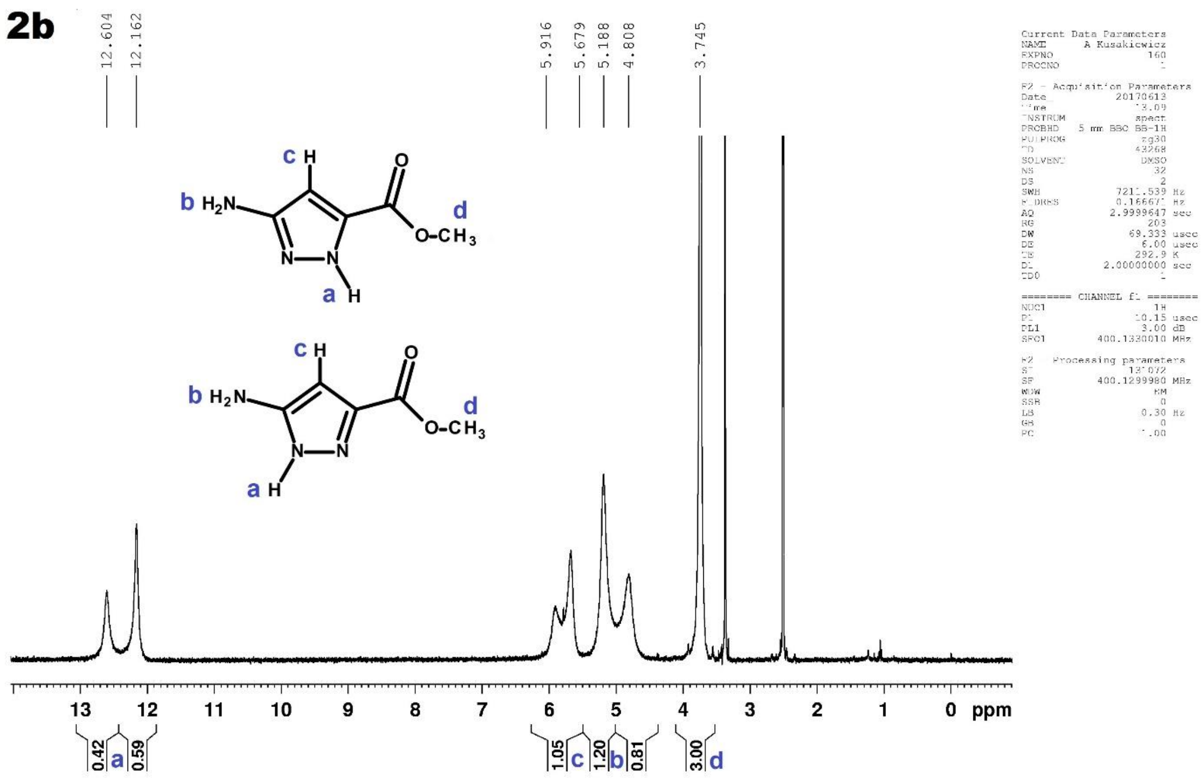


**Figure 14S.** $^{1}H$ NMR spectrum of methyl 5(3)-amino-1*H*-pyrazole-3(5)-carboxylate (**2b**) in DMSO-$d_6$.

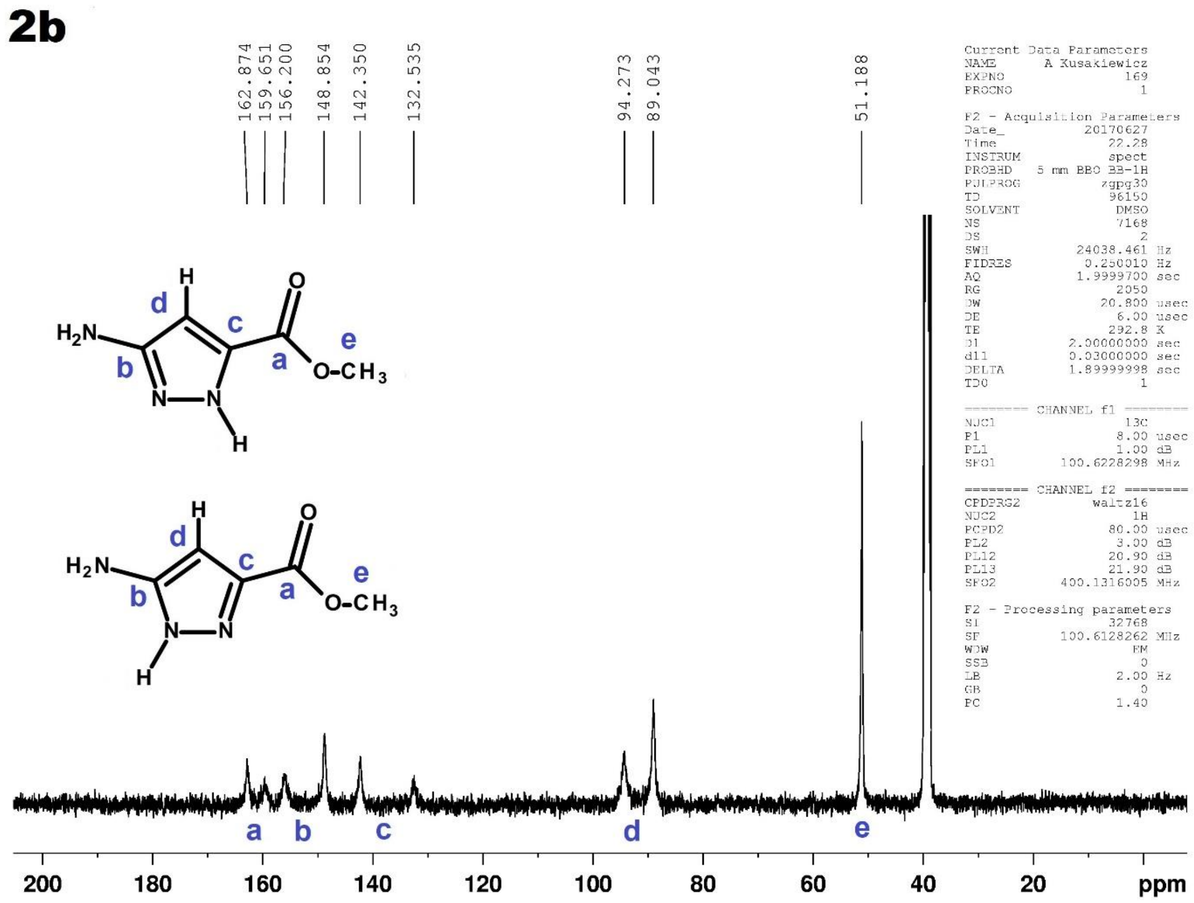


**Figure 15S.** $^{13}$C NMR spectrum of methyl 5(3)-amino-1*H*-pyrazole-3(5)-carboxylate (**2b**) in DMSO-$d_6$.

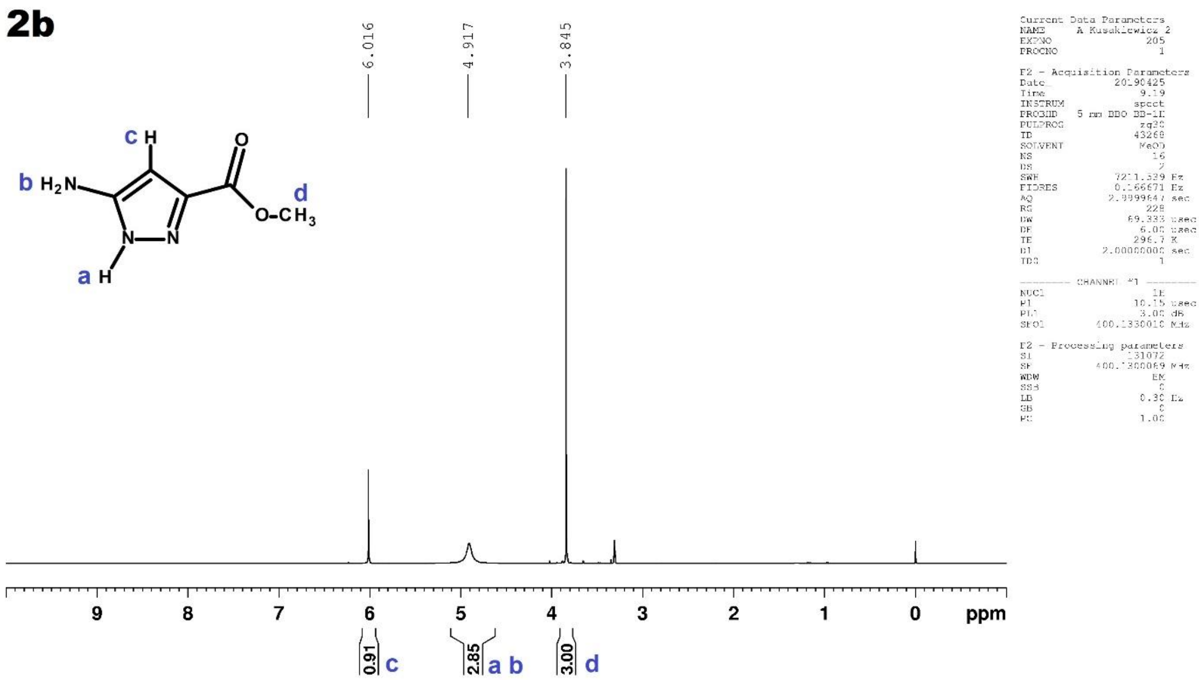


**Figure 16S.** $^{1}$H NMR spectrum of methyl 5(3)-amino-1*H*-pyrazole-3(5)-carboxylate (**2b**) in $CD_3OD$.

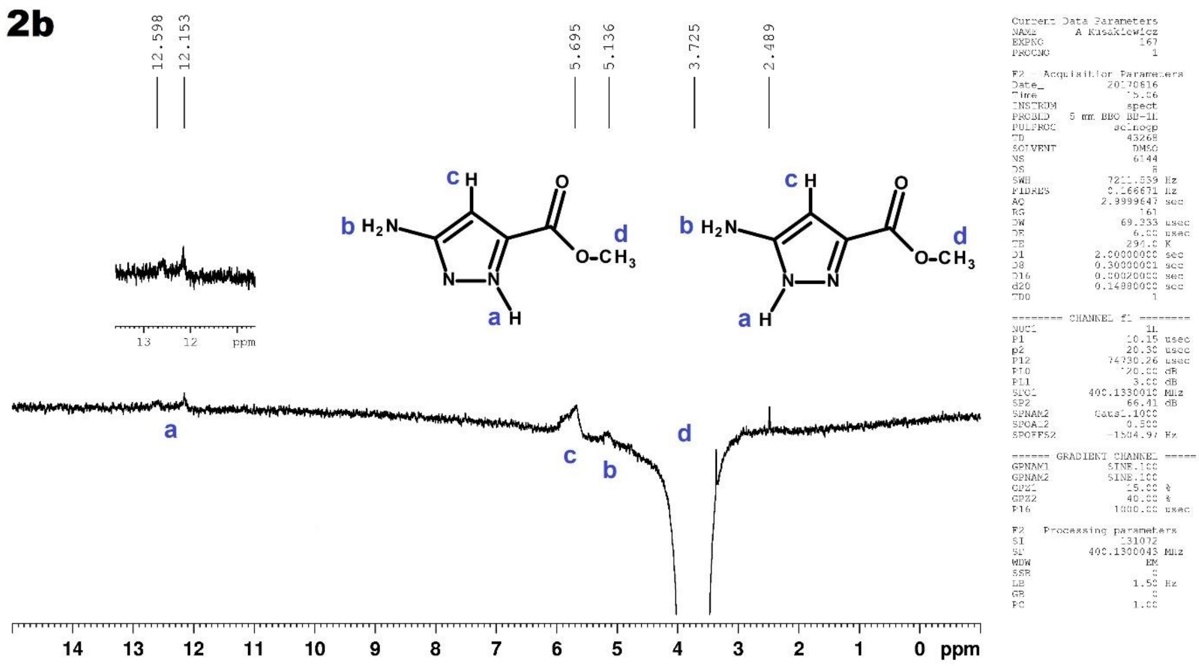


**Figure 17S.** NOE spectrum of methyl 5(3)-amino-1*H*-pyrazole-3(5)-carboxylate (**2b**) in DMSO-d$_6$.

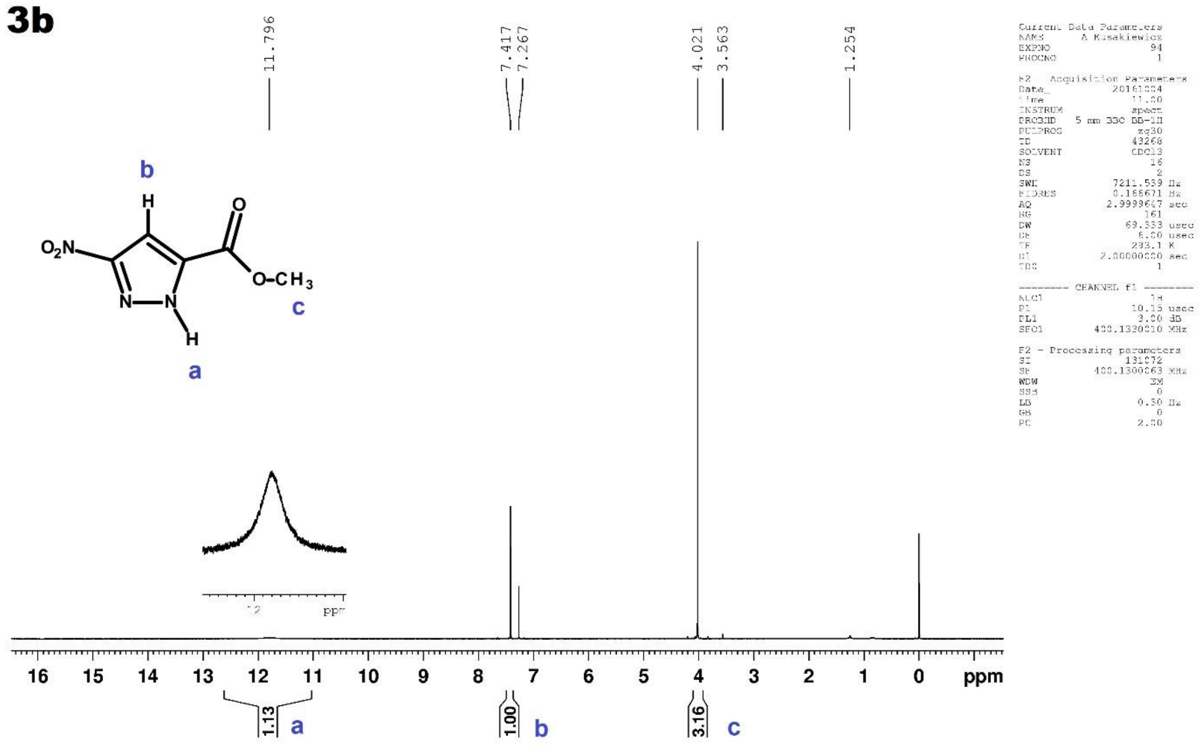


**Figure 18S.** $^{1}$H NMR spectrum of methyl 3-nitro-1*H*-pyrazole-5-carboxylate (**3b**) in CDCl$_3$.

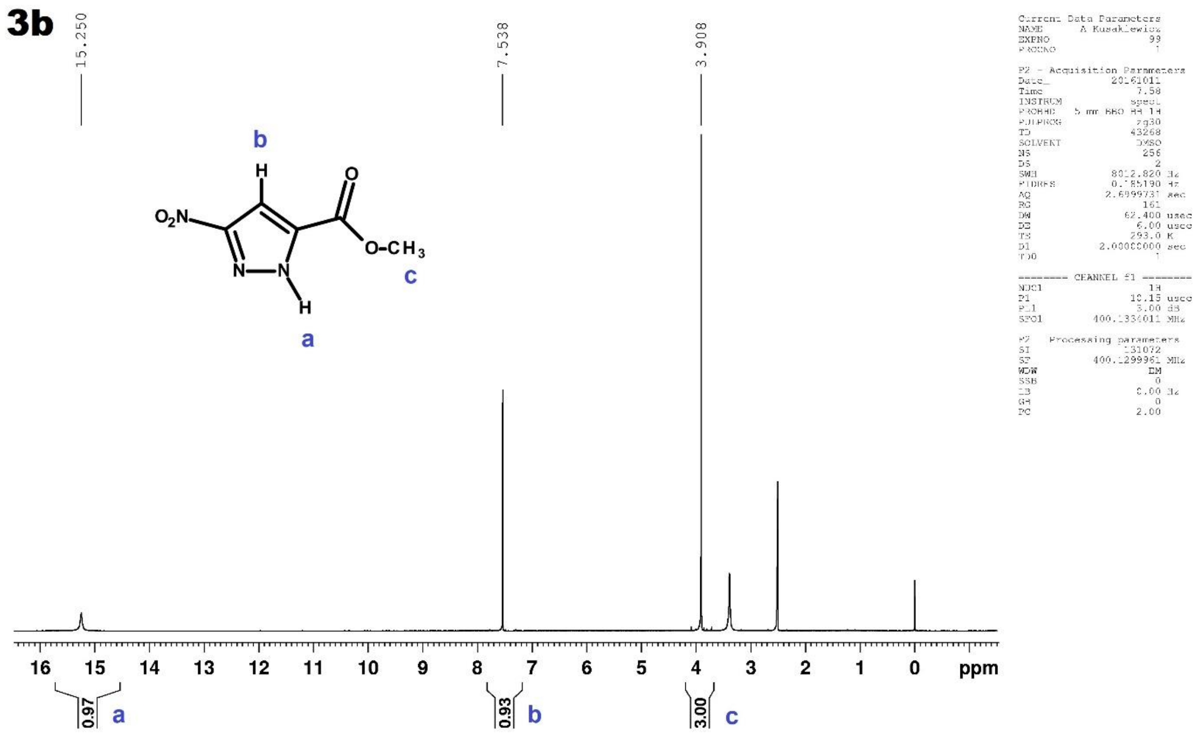


**Figure 19S.** $^1$H NMR spectrum of methyl 3-nitro-1*H*-pyrazole-5-carboxylate (**3b**) in DMSO-$d_6$.

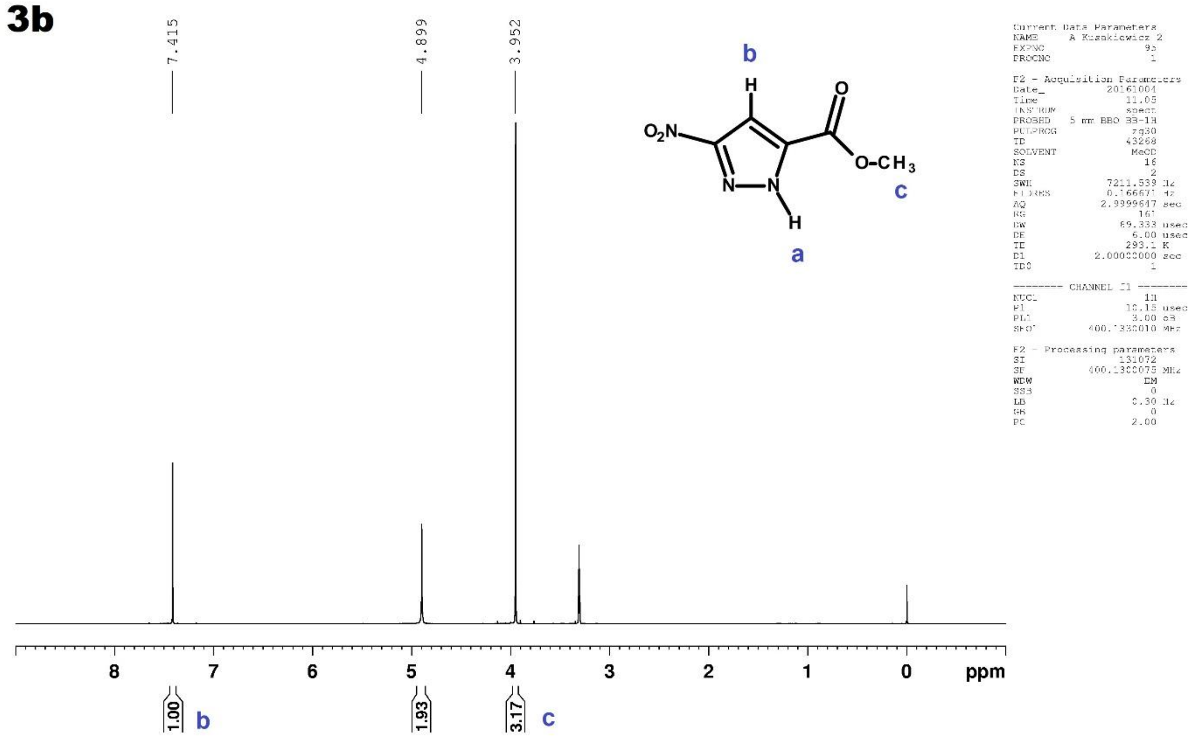


**Figure 20S.** $^1$H NMR spectrum of methyl 3-nitro-1*H*-pyrazole-5-carboxylate (**3b**) in $CD_3OD$.

**3b**

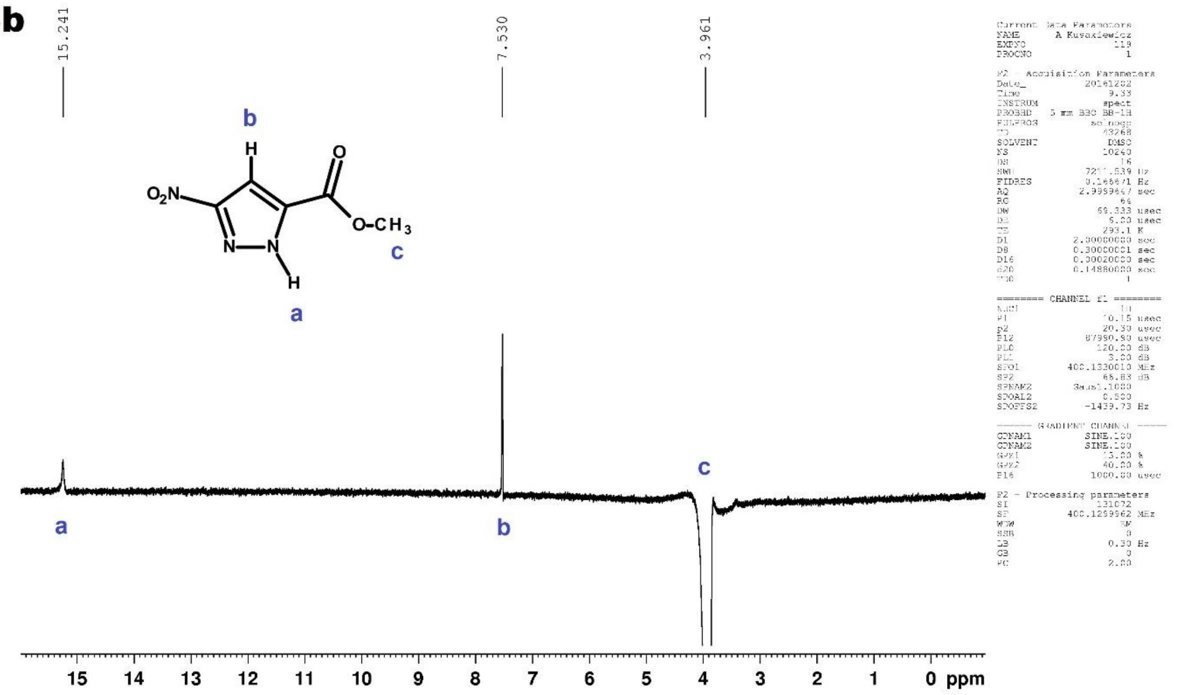


**Figure 21S.** NOE spectrum of methyl 3-nitro-1*H*-pyrazole-5-carboxylate (**3b**) in DMSO-$d_6$.

**Supplementary Materials**

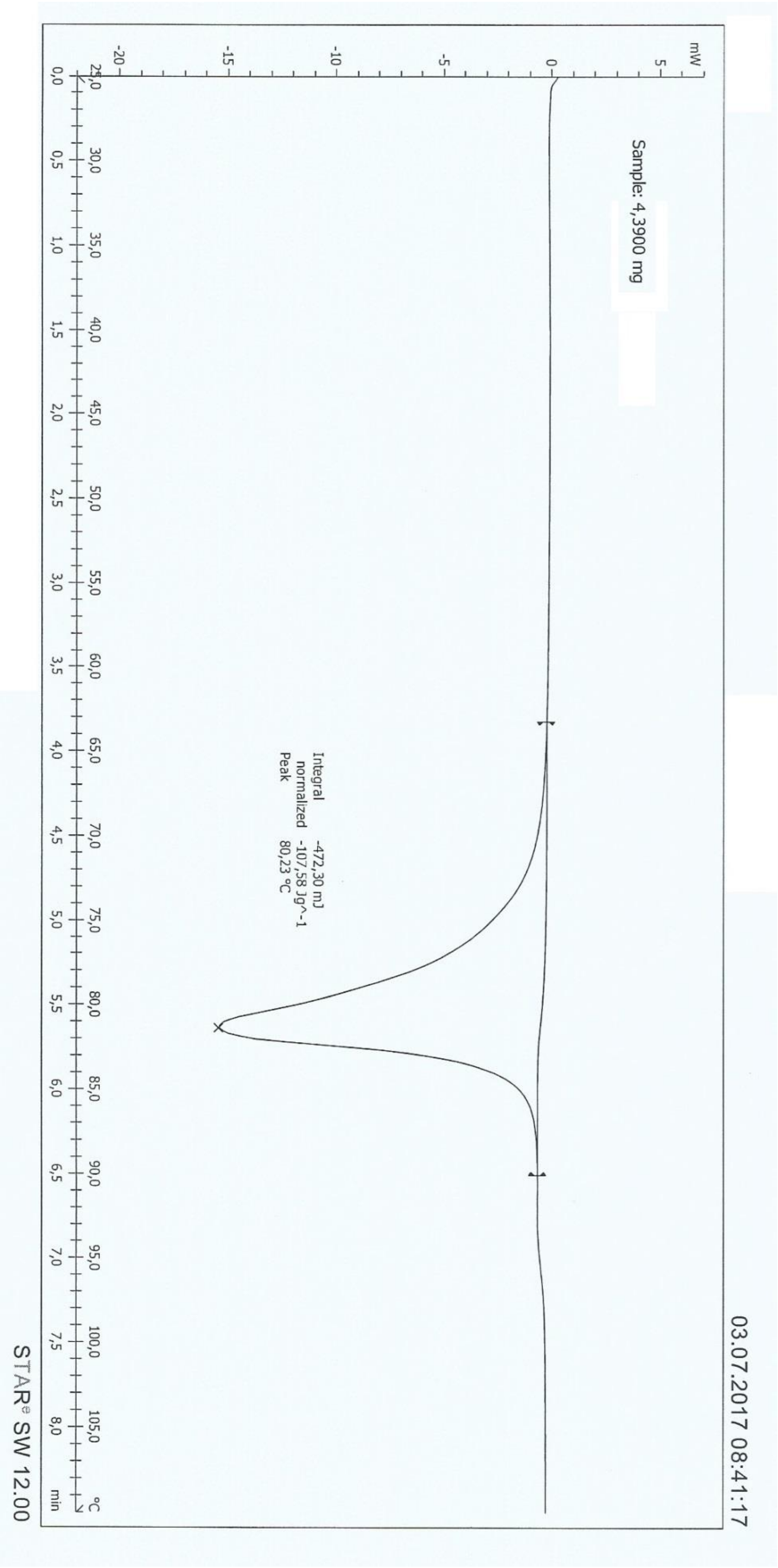


**Figure 22S.** DSC of ethyl 5-methyl-1H-pyrazole-3-carboxylate (**1b**)

**Supplementary Materials**

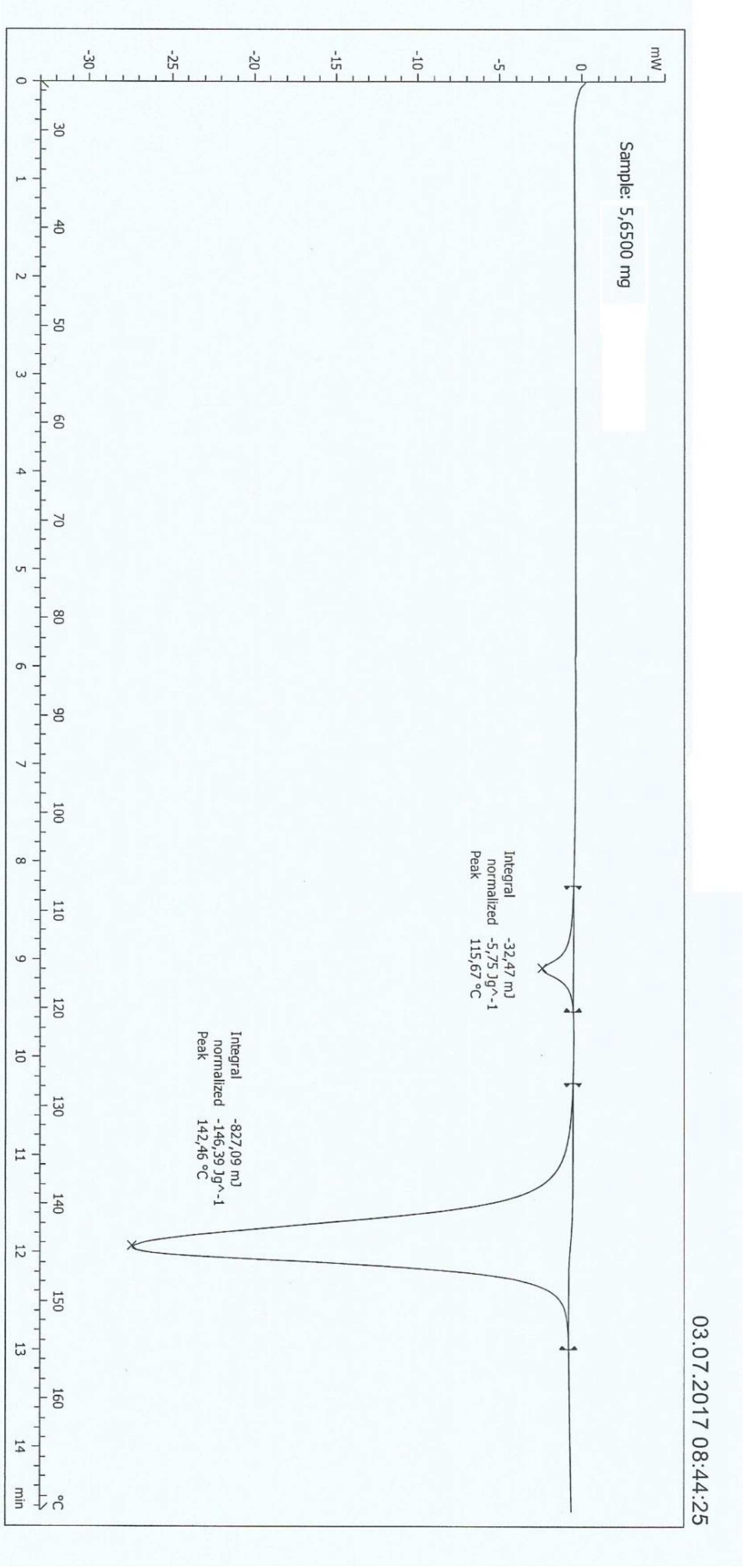


**Figure 23S.** DSC of methyl 5(3)-amino-1H-pyrazole-3(5)-carboxylate (**2b**)

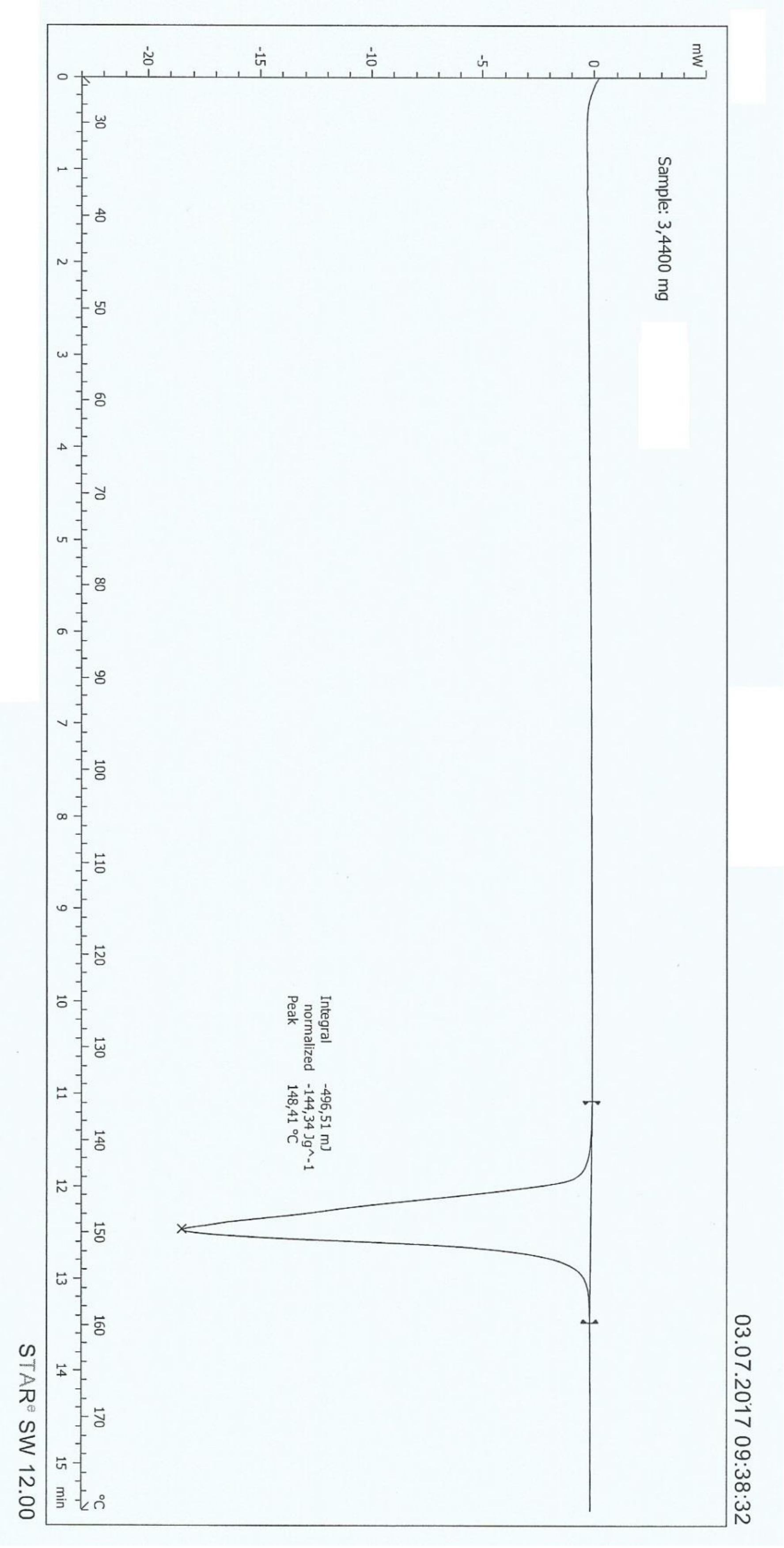


**Figure 24S.** DSC of methyl 3-nitro-1*H*-pyrazole-5-carboxylate (**3b**).
